# Distribution-Dependent Weighted Union Bound †

**DOI:** 10.3390/e23010101

**Published:** 2021-01-12

**Authors:** Luca Oneto, Sandro Ridella

**Affiliations:** 1 Department of Computer Science, Bioengineering, Robotics and Systems Engineering, University of Genoa, Via Opera Pia 11a, 16145 Genova, Italy; 2 Department of Biophysical and Electronic Engineering, University of Genoa, Via Opera Pia 11a, 16145 Genova, Italy; sandro.ridella@unige.it

**Keywords:** union bound, weighted union bound, distribution-dependent weights, statistical learning theory, finite number of hypothesis

## Abstract

In this paper, we deal with the classical Statistical Learning Theory’s problem of bounding, with high probability, the true risk R(h) of a hypothesis *h* chosen from a set H of *m* hypotheses. The Union Bound (UB) allows one to state that $P\left\{L\left(\hat{R}\left(h\right),\delta q_{h}\right)\leq R\left(h\right)\leq U\left(\hat{R}\left(h\right),\delta p_{h}\right)\right\}\geq 1-\delta$ where $\hat{R}\left(h\right)$ is the empirical errors, if it is possible to prove that $P\{R\left(h\right)\geq L\left(\hat{R}\left(h\right),\delta \right)\}\geq 1-\delta$ and $P\{R\left(h\right)\leq U\left(\hat{R}\left(h\right),\delta \right)\}\geq 1-\delta$, when *h*, $q_{h}$, and $p_{h}$ are chosen before seeing the data such that $q_{h},p_{h}\in \left[0,1\right]$ and $\sum_{h\in H}\left(q_{h}+p_{h}\right)=1$. If no *a priori* information is available $q_{h}$ and $p_{h}$ are set to 12m, namely equally distributed. This approach gives poor results since, as a matter of fact, a learning procedure targets just particular hypotheses, namely hypotheses with small empirical error, disregarding the others. In this work we set the $q_{h}$ and $p_{h}$ in a distribution-dependent way increasing the probability of being chosen to function with small true risk. We will call this proposal Distribution-Dependent Weighted UB (DDWUB) and we will retrieve the sufficient conditions on the choice of $q_{h}$ and $p_{h}$ that state that DDWUB outperforms or, in the worst case, degenerates into UB. Furthermore, theoretical and numerical results will show the applicability, the validity, and the potentiality of DDWUB.

## 1. Introduction

Statistical learning theory [1,2,3,4] deals with the problem of understanding and estimating the performance of a statistical learning procedure. The goal is to better understand the factors that influence its behavior and to suggest ways to improve it. Although asymptotic analysis is a crucial first step in this direction, finite sample error bounds are of more value as they allow the design of model selection procedures [5,6,7]. These error bounds typically have the following form: with high probability, the generalization error of the selected hypothesis, chosen in a space of possible ones, is bounded by an empirical estimate of the generalization error plus a penalty term which depends on the size of the hypothesis space and the number of samples available. The latter term basically considers that the learning procedure selects a hypothesis in a set of possible ones based on the available data. Every data-dependent choice implies a risk, and the penalty term is exactly the measure of this risk. When the hypothesis space is composed of an arbitrary finite number of hypothesis, and no additional information is provided, the evaluation of the total risk is usually made with the Union Bound (UB) [2,7,8]. The UB is an ubiquitous building block in statistical learning theory and is exploited in many context and in many different ways to derive the final result: in the Vapnik–Chervonenkis theory [2], in the Rademacher Complexity theory [9,10], in the Algorithmic Stability theory [11], in the Compression Bound [12], in the PAC-Bayes theory [13], and more recently in the Differential Privacy theory [14].

Let us consider the classical binary classification framework (The extension to the general supervised learning characterized by bounded loss functions will be discussed later during the presentation.). Let X be the input space and Y={−1,+1} be the set of binary output labels. Let $D_{n}=\left\{\left(X_{1},Y_{1}\right),\ldots ,\left(X_{n},Y_{n}\right)\right\}$, where $X_{i}\in X$ and $Y_{i}\in Y$, ∀i∈{1,⋯,n}, be a sequence of $n\in N^{*}$ samples drawn independently from an unknown probability distribution μ over X×Y. Let us consider a hypothesis h:X→Y chosen from a finite set H of possible hypotheses of cardinality $m\in N^{*}$ such that $H=\{h_{i}:i\in I\}$ where I={1,⋯,m}. The error of *h* in approximating P{Y|X} is measured by a prescribed loss function ℓ:Y×Y→R. Since we are dealing with binary classification problems the most natural choice is the loss function which counts the number of errors ℓ(h(X),Y)=𝟙{Y≠h(X)}∈{0,1}. The generalization error of *h* is defined as

$$\begin{array}{c} R(h)=E\{\ell (h(X),Y)\}\in [0,1]. \end{array}$$

Since the probability measure μ is usually unknown, the generalization error cannot be computed; however, we can compute the empirical error

$$\begin{array}{c} \hat{R}\left(h\right)=\frac{1}{n}\sum_{i=1}^{n}\ell \left(h\left(X_{i}\right),Y_{i}\right)\in \left[0,1\right]. \end{array}$$

If the choice of h∈H does not depend on $D_{n}$, namely if we want to bound the generalization error of a single hypothesis in the hypothesis space chosen before seeing the data, it is possible to prove that (Please note that for simplicity, we will refer to R(h) and $\hat{R}\left(h\right)$ with *R* and $\hat{R}$ respectively, when it is clear from the context.)

$$\begin{array}{c} P\left\{R\geq L(\hat{R},\delta )\right\}\geq 1-\delta ,\;P\left\{R\leq U(\hat{R},\delta )\right\}\geq 1-\delta , \end{array}$$

where δ∈(0,1) while L and U are respectively lower and upper bounds of the generalization error (see, for example, [15,16,17]).

Since the generalization error cannot be smaller than zero or larger than one consequently we have that $L\left(\hat{r},\delta \right)\in \left[0,1\right)$ and $U\left(\hat{r},\delta \right)\in \left(0,1\right]$$\forall \hat{r}\in \left[0,1\right]$ and ∀δ∈(0,1) [15]. When, instead of [15,16], or similar results, are exploited it is necessary to truncate them.

In general, the choice of h∈H does depend on $D_{n}$: in this case we must estimate the risk due to this data-dependent choice.

As an example, common practice for choosing h∈H based on $D_{n}$ is to choose the hypothesis with minimum empirical error

$$\begin{array}{c} \arg\min_{h\in H}\hat{R}\left(h\right), \end{array}$$

and this approach is called Empirical Risk Minimization [2,18], but others possibilities exist such as the Structural Risk Minimization [2,19,20], or the penalized (regularized) Empirical Risk Minimization [21,22,23].

To guarantee a prescribed confidence level, or risk, of the chosen hypothesis, the UB can be applied. The UB can be expressed in two forms (Please note that for simplicity, we will refer to $R(h_{i})$ and $\hat{R}\left(h_{i}\right)$ with $R_{i}$ and ${\hat{R}}_{i}$ respectively, when it is clear from the context.) [8]: a simplified version (Theorem 1) and a generalized version (Theorem 2).

**Theorem** **1** (Simple UB). *The following bounds hold*

$$\begin{array}{c} P\left\{L\left({\hat{R}}_{i},\frac{\delta }{2m}\right)\leq R_{i}\leq U\left({\hat{R}}_{i},\frac{\delta }{2m}\right)\;\forall i\in I\right\}\geq 1-\delta . \end{array}$$

**Theorem** **2** (Generalized UB). *Let $q\left(h_{i}\right)\in \left(0,1\right)$ and $p\left(h_{i}\right)\in \left(0,1\right)$ be some weight associated with $h_{i}$ with i∈I before seeing the data (Please note that for simplicity, we will refer to $q(h_{i})$ and $p(h_{i})$ with $q_{i}$ and $p_{i}$ respectively, when it is clear from the context.) and such that $\sum_{i\in I}\left(q_{i}+p_{i}\right)=1$, then the following bounds hold*

$$\begin{array}{c} P\left\{L\left({\hat{R}}_{i},\delta q_{i}\right)\leq R_{i}\leq U\left({\hat{R}}_{i},\delta p_{i}\right)\;\forall i\in I\right\}\geq 1-\delta . \end{array}$$

Theorem 1 is a special case of Theorem 2 when qi=pi=12m∀i∈{1,⋯,m}.

Theorem 2 introduces a weight for each risk associated with each choice. Weighting more the risk associated with useful choices leads to tighter bounds on the generalization error of hypotheses that will be selected by the algorithm (hypotheses characterized by small empirical error) and looser estimates over the others (hypotheses characterized by high empirical error). Unfortunately, this approach is mainly theoretical since the weights must be chosen before seeing the data and consequently we cannot set them without an *a priori* knowledge about the problem. Finally, Theorem 2 does not propose any solution for the choice of these weights.

For this reason, in this work, we propose a Distribution-Dependent Weighted UB (DDWUB) where the weights depend on some parameters of the distribution which generated them, extending our preliminary work [24]. In particular, we define a set of functions $f_{i}^{p}:R^{m}\to R$ and $f_{i}^{q}:R^{m}\to R$ with i∈I such that

$$\begin{array}{cc} \; & q_{i}=f_{i}^{q}\left(R_{1},\cdots ,R_{m}\right),p_{i}=f_{i}^{p}\left(R_{1},\cdots ,R_{m}\right)\in \left(0,1\right),\;\forall i\in I,\\ \; & \sum_{i\in I}\left(f_{i}^{q}\left(R_{1},\cdots ,R_{m}\right)+f_{i}^{p}\left(R_{1},\cdots ,R_{m}\right)\right)=1. \end{array}$$

Please note that $f_{i}^{q},f_{i}^{p}$ with i∈I are quite general and are data independent (Please note that in the framework of the paper (binary classification with a loss function which counts the number of misclassified samples), the generalization error is the only parameter of the distribution which is a Binomial). It is surely possible to consider even more general data independent functions for defining the weights, but we think that our definition is general enough to contemplate a wide variety of cases.

At this point the proposed DDWUB for bounding the generalization error of a hypothesis chosen from a finite set of possible ones can be stated.

**Theorem** **3.** *If $\forall r_{1},\cdots ,r_{m}\in \left[0,1\right]$*

$$\begin{array}{cc} \; & f_{i}^{q}\left(r_{1},\cdots ,r_{m}\right),f_{i}^{p}\left(r_{1},\cdots ,r_{m}\right)\in \left(0,1\right),\;\forall i\in I,\\ \; & \sum_{i\in I}\left(f_{i}^{q}\left(r_{1},\cdots ,r_{m}\right)+f_{i}^{p}\left(r_{1},\cdots ,r_{m}\right)\right)=1, \end{array}$$

*then the following bound holds*

$$\begin{array}{c} P\left\{L\left({\hat{R}}_{i},\delta f_{i}^{q}\left(R_{1},\cdots ,R_{m}\right)\right)\leq R_{i}\leq U\left({\hat{R}}_{i},\delta f_{i}^{p}\left(R_{1},\cdots ,R_{m}\right)\right)\;\forall i\in I\right\}\geq 1-\delta . \end{array}$$

The proof is a direct consequence of Theorem 2.

DDWUB allow the binding of the generalization error of each hypothesis in the space of hypotheses but, to prove that the DDWUB outperforms the UB, we will require some sufficient conditions that L, U, $f_{i}^{q}$, and $f_{i}^{p}$ with i∈I must satisfy. These sufficient conditions define a class of functions and an open problem would be to find special and simpler classes of functions which satisfy them.

Nevertheless, we will show that it is possible to find simple classes of functions which satisfy these conditions. For example, if one is interested in having tighter upper bound of the generalization error of the empirical minimizer DDWUB suggest combining classical L and U, such as [15] or [16], and set

$$\begin{array}{c} f_{i}^{q}\left(R_{1},\cdots ,R_{m}\right)=\frac{1}{2m},\;f_{i}^{p}\left(R_{1},\cdots ,R_{m}\right)=\frac{1}{2}\frac{e^{-\gamma \max[\theta ,R_{i}]}}{\sum_{j\in I}e^{-\gamma \max[\theta ,R_{j}]}},\;\forall i\in I, \end{array}$$

with particular values of γ∈[0,∞) and θ∈[0,1].

As a last remark we would like to note that all our results easily extend to multiclass classification problems and regression problems if the loss function is bounded.

DDWUB is a distribution-dependent form of the UB analogously to the Computable Shell Decomposition Bounds (CSDB) [20]. The CSDB splits the hypothesis space in shells based on the generalization error of each hypothesis and, instead of taking into account the risk of each hypothesis in the space, show that it is possible to just take into account the risk of choosing one shell and the risk associated with each hypothesis in the shell. This allows, for example, to not consider hypotheses with high generalization error. The CSDB show also how to estimate the size of these shells based on the histogram of the empirical errors.

DDWUB takes inspiration from several works in the field. The first idea, which is also a driver of the CSDB, is that during any learning procedure the hypotheses with high error will be never taken into account and consequently we should not pay the risk for those hypotheses [10]. The second idea is that since we do not know the true error of the hypotheses but just its empirical one, we should discard those hypotheses for which the estimated confidence intervals do not overlap [25] with the ones of the hypothesis of minimal training error. The third idea is that since there is no supporting theory for discarding the hypothesis with non-overlapping confidence intervals, we should weight differently the risk associated with each hypothesis based on their true error analogously to what is done in the field of multiple hypotheses testing [26]. The fourth idea is that other researchers have shown that a distribution-dependent weighting strategy can be performed without the actual knowledge of the distribution [27]. DDWUB combines all these ideas and improves both on the UB and the CSDB.

DDWUB applies to finite hypotheses spaces and surely more sophisticated techniques, such as Local Vapnik–Chervonenkis [28] or the Local Rademacher Complexity [10], can be employed and can sometimes result in tighter bounds. However, insight into finite classes remains quite useful [20,29]. Finite class analysis can be exploited for as a pedagogical tool. Finite class analysis can teach new directions in which to look for the development and evolution of more sophisticated bounds. Finite class analysis can be useful for model selection purposes (e.g., selecting the most suitable hypothesis space, or set of hyperparameters, or algorithm). Finite class analysis can be useful when the models are represented with limited number of bits because of the constants involved in the bounds.

The rest of the paper is organized as follows. Section 2 presents the DDWUB in a simplified setting. In Section 3 we present the DDWUB in a generalized setting, we derive the sufficient conditions which state when DDWUB improves over the UB, we will show that it is possible to find simple classes of functions which satisfy these conditions, and we will make the connection between our results and the ones of [25]. Section 4 reports a comparison between DDWUB and the UB by means of closed form results. Section 5 reports a comparison between DDWUB and the UB by means of an extensive set of numerical results. Section 6 compares DDWUB with CSDB by means of an extensive set of numerical results. Section 7 shows the applicability and the potentiality of DDWUB. Section 8 concludes the paper. In the Appendices known results, proof, and technicalities (See in Appendix A, Appendix B and Appendix C) are reported for completeness.

## 2. Distribution-Dependent Weighted Union Bound: Simplified Setting

Let us consider Theorem 3 and the bound proposed by [16] recalled by Theorem A1 in Appendix A. Let us also suppose, for simplicity, that we are interested in upper bounding the generalization error of the empirical risk minimizer (Extensions will be discussed at the end of this section). In this setting it is possible to state our DDWUB.

**Corollary** **1.** *If*

$$\begin{array}{c} f_{i}\left(r_{1},\cdots ,r_{m}\right)=\frac{e^{-\gamma \max[\theta ,r_{i}]}}{\sum_{j\in I}e^{-\gamma \max[\theta ,r_{j}]}},\;\forall i\in I, \end{array}$$

*with γ∈[0,∞) and θ∈[0,1] then the following bound holds*

$$\begin{array}{c} P\left\{\max\left[0,{\hat{R}}_{i}-\sqrt{\frac{\log\left(\frac{2m}{\delta }\right)}{2n}}\right]\leq R_{i}\leq \min\left[1,{\hat{R}}_{i}+\sqrt{\frac{\log\left(\frac{2}{\delta f_{i}\left(R_{1},\cdots ,R_{m}\right)}\right)}{2n}}\right]\;\forall i\in I\right\}\geq 1-\delta . \end{array}$$

Corollary 1 is a direct consequence of Theorems 3 and A1.

The choice of the weights takes inspiration from the work of [27] which proposed, in the context of the PAC-Bayes theory, a distribution-dependent method for assigning an a priori distribution over a set of hypotheses to give a higher probability to the hypothesis with small generalization error. This method has been shown to possess interesting theoretical properties [30,31] and to be also quite effective in practical applications [32].

Since we are interested in choosing and bounding the generalization error of the empirical minimizer, let us define

$$\begin{array}{c} i^{*}=\arg\min_{i\in I}{\hat{R}}_{i}. \end{array}$$

This approach is analogous to Page’s criterion [33], which was designed as a process inspection scheme to detect deviations in average in only one direction (one-sided) in a stochastic process.

In Corollary 1, γ acts as a weighting factor. The larger is γ the larger are the weights of the risks associated with hypotheses with small empirical error and the smaller are the weights of the risks associated with hypotheses with large empirical error. For γ→∞ we have that (For simplicity, we assume in this statement that the empirical minimizer is unique.) $p_{i^{*}}\to 1$ and $p_{i}\to 0$$\forall i\in I\backslash i^{*}$. The smaller is γ the less is the difference between the weights of the risks. For γ→0 we have that pi=1m∀i∈I.

In Corollary 1, θ, instead, acts as a protection against the fact that the empirical error is measured over a finite number of samples and, if the sample size is small, hypotheses with a small difference in the empirical error are indistinguishable. In other words, the weights depend on unknown parameters of the data generating distribution, then we will have to estimate them and since the number of sample is finite these estimates will not allow us to distinguish hypotheses which show similar empirical error. For this reasons, θ gives the same the weight to the risks associated with hypotheses with small empirical error.

The values of γ and θ must be set in a particular way to be sure that DDWUB improves over the UB. In particular

- Lemma 1 shows that to upper bound the generalization error of the empirical risk minimizer based on DDWUB of Corollary 1 we must solve an optimization problem;
- Lemmas 2 and 3 show that for particular values of γ the solution is unique and can be found by simply search for the fixed point of a simple function;
- Theorem 4 and Lemma 4 show that for particular values of θ it is possible to prove that DDWUB is tighter than, or in the worst case as tight as, the UB.

Thanks to Corollary 1 we can state the following lemma.

**Lemma** **1.** *Under the same conditions of Corollary 1 if*

$$\begin{array}{c} i^{*}=\arg\min_{i\in I}{\hat{R}}_{i}, \end{array}$$

*then we can state that following bound holds*

$$\begin{array}{cc} \; & R_{i^{*}}\leq \max_{r_{1},\cdots ,r_{m}}\;r_{i^{*}}\\ \; & s.t.\;\max\;\;\left[0,{\hat{R}}_{i}-\sqrt{\frac{\log\left(\frac{2m}{\delta }\right)}{2n}}\right]\;\;\leq r_{i}\leq \min\;\;\left[1,{\hat{R}}_{i}+\sqrt{\frac{\log\left(\frac{2\sum_{j\in I}e^{-\gamma \max[\theta ,r_{j}]}}{\delta e^{-\gamma \max[\theta ,r_{i}]}}\right)}{2n}}\right]\;\;,\forall i\in I. \end{array}$$

Lemma 1 can be further simplified as follows.

**Lemma** **2.** *Under the same conditions of Lemma 1 the following bound holds*

$$\begin{array}{cc} R_{i^{*}}\leq \max_{r_{i^{*}}}\; & r_{i^{*}}\\ s.t.\; & r_{i^{*}}\leq \min\left[1,{\hat{R}}_{i^{*}}+\sqrt{\frac{\log\left(\frac{2\sum_{j\in I}e^{-\gamma \max[\theta ,r_{j}]}}{\delta e^{-\gamma \max[\theta ,r_{i^{*}}]}}\right)}{2n}}\right], \end{array}$$

where $r_{i}=\max\left[0,{\hat{R}}_{i}-\sqrt{\frac{\log\left(\frac{2m}{\delta }\right)}{2n}}\right]$$\forall i\in I\backslash i^{*}$.

The proof can be found in Appendix B.

Please note that the optimization problem of Lemma 2 can be further simplified noting that for particular values of γ, the solution of the optimization problem is unique.

**Lemma** **3.** *Under the same conditions of Lemma 2 if*

$$\begin{array}{c} \gamma \leq 2\sqrt{n}, \end{array}$$

the solution of the optimization problem of Lemma 2 exists, it is unique, and it is the fixed point $r_{i^{*}}^{*}$ of the following function of $r_{i^{*}}$

$$\begin{array}{c} r_{i^{*}}=\min\left[1,{\hat{R}}_{i^{*}}+\sqrt{\frac{\log\left(\frac{2\sum_{j\in I}e^{-\gamma \max[\theta ,r_{j}]}}{\delta e^{-\gamma \max[\theta ,r_{i^{*}}]}}\right)}{2n}}\right] \end{array}$$

where $r_{i}=\max\left[0,{\hat{R}}_{i}-\sqrt{\frac{\log\left(\frac{2m}{\delta }\right)}{2n}}\right]$$\forall i\in I\backslash i^{*}$.

The proof can be found in Appendix B.

Please note that to find the fixed point defined in Lemma 3 a simple bisection method can be applied.

For particular values of θ, it is possible to state that DDWUB is tighter than, or in the worst case as tight as, the UB.

**Theorem** **4.** *Under the same conditions of Lemma 3 if*

$$\begin{array}{c} \theta \geq \min\left[1,{\hat{R}}_{i^{*}}+\sqrt{\frac{\log\left(\frac{2m}{\delta }\right)}{2n}}\right], \end{array}$$

*then*

$$\begin{array}{c} r_{i^{*}}^{*}\leq \min\left[1,{\hat{R}}_{i^{*}}+\sqrt{\frac{\log\left(\frac{2m}{\delta }\right)}{2n}}\right]. \end{array}$$

The proof can be found in Appendix B.

The problem of the θ defined in Theorem 4 is that it is data-dependent since we do not know $i^{*}$ before seeing the data. For this reason, the following lemma suggests a data independent threshold of θ which satisfies the conditions of Theorem 4.

**Lemma** **4.** *Under the same conditions of Theorem 4*

$$\begin{array}{c} \min\left[1,{\hat{R}}_{i^{*}}+\sqrt{\frac{\log\left(\frac{2m}{\delta }\right)}{2n}}\right]\leq \min\left[1,\min\left[R_{1},\cdots ,R_{m}\right]+2\sqrt{\frac{\log\left(\frac{2m}{\delta }\right)}{2n}}\right]. \end{array}$$

The proof can be found in Appendix B.

Lemma 4 provides us a method for finding a $\theta \geq U\left({\hat{R}}_{i^{*}},\frac{\delta }{2m}\right)$ in a data independent way by setting

(1) $$\begin{array}{c} \theta =\min\left[1,\min\left[R_{1},\cdots ,R_{m}\right]+2\sqrt{\frac{\log\left(\frac{2m}{\delta }\right)}{2n}}\right]. \end{array}$$

By finding the $r_{i^{*}}^{*}$ for all possible values of θ and then by selecting the largest one which satisfies Equation (1) we have the results of our DDWUB.

Please note that the above-mentioned result easily extends to the whole supervised learning framework, until a bounded loss function is employed, since the inequality proposed by [16] cover this case.

Following the same argument described in this section it is possible to derive the DDWUB for lower bounding the generalization error of the empirical risk minimizer by simply setting in Theorem 3

$$\begin{array}{c} f_{i}^{q}\left(R_{1},\cdots ,R_{m}\right)=\frac{1}{2}\frac{e^{-\gamma \min[\theta ,1-R_{i}]}}{\sum_{j\in I}e^{-\gamma \min[\theta ,1-R_{j}]}},\;f_{i}^{p}\left(R_{1},\cdots ,R_{m}\right)=\frac{1}{2m},\;\forall i\in I. \end{array}$$

Finally, it is possible to derive the DDWUB for upper and lower bounding the generalization error of the empirical risk minimizer by simply setting in Theorem 3

$$\begin{array}{cc} \; & f_{i}^{q}\left(R_{1},\cdots ,R_{m}\right)=\frac{1}{2}\frac{e^{-\gamma \min[\theta ,1-R_{i}]}}{\sum_{j\in I}e^{-\gamma \min[\theta ,1-R_{j}]}},\;\forall i\in I,\\ \; & f_{i}^{p}\left(R_{1},\cdots ,R_{m}\right)=\frac{1}{2}\frac{e^{-\gamma \max[\theta ,R_{i}]}}{\sum_{j\in I}e^{-\gamma \max[\theta ,R_{j}]}},\;\forall i\in I. \end{array}$$

**Example** **1.** *Before presenting DDWUB in the general setting we would like to show an application of DDWUB in the simplified setting. Let us consider the case when (More general examples can be derived, and we will do it later with both closed form and numerical results, but here we want to keep the presentation as simple as possible.)*

$$\begin{array}{c} {\hat{R}}_{1}={\hat{R}}_{2}=0,\;{\hat{R}}_{3}={\hat{R}}_{4}=\cdots ={\hat{R}}_{m}=\nu ,\;\nu \in \left\{\frac{1}{n},\frac{2}{n},\cdots ,1\right\}. \end{array}$$

*Let us set $\gamma =2\sqrt{n}$ (see Lemma 3) and note that to upper bound the function with the smallest empirical error (i.e., the one corresponding to ${\hat{R}}_{1}$) we have that DDWUB states that*

$$\begin{array}{c} \left\{\begin{array}{c} r_{1}=\sqrt{\frac{\ln\left(\frac{2\sum_{i=1}^{m}e^{-2\sqrt{n}\max\left[\theta ,r_{i}\right]}}{\delta e^{-2\sqrt{n}\max\left[\theta ,r_{1}\right]}}\right)}{2n}}\\ r_{2}=0\\ r_{3}=r_{4}=\cdots =\nu -\sqrt{\frac{\ln\left(\frac{2m}{\delta }\right)}{2n}}\\ \theta =\min\left[1,\min\left[r_{1},\cdots ,r_{m}\right]+2\sqrt{\frac{\log\left(\frac{2m}{\delta }\right)}{2n}}\right]. \end{array}\right.. \end{array}$$

*Please note that for a finite but large enough value of n*

$$\begin{array}{c} \min\left[r_{1},\cdots ,r_{m}\right]=0\to \theta =2\sqrt{\frac{\ln\left(\frac{2m}{\delta }\right)}{2n}}. \end{array}$$

*Thanks to the theory of DDWUB (see Lemma 4) we can state that*

$$\begin{array}{c} r_{1}^{*}\leq \theta . \end{array}$$

*Let us note that if*

$$\begin{array}{c} m<\frac{\delta e^{2n{\left(\frac{\nu }{2}\right)}^{2}}}{2}, \end{array}$$

*then*

$$\begin{array}{c} r_{3}=\cdots =r_{m}>\theta . \end{array}$$

*Then we can easily state that*

$$\begin{array}{c} \lim_{n\to \infty }\frac{2\sum_{i=1}^{m}e^{-2\sqrt{n}\max\left[\theta ,r_{i}\right]}}{\delta e^{-2\sqrt{n}\max\left[\theta ,r_{1}\right]}}=\frac{4}{\delta }, \end{array}$$

*which means that all the hypothesis in the space with $\hat{R}\neq 0$, if m<δe2nν222, are not taken into account, asymptotically, in estimating the upper bound of the hypothesis with the smaller error with DDWUB.*

## 3. Distribution-Dependent Weighted Union Bound: General Setting

In this section, we will derive the sufficient conditions for stating that DDWUB is tighter than, or in the worst case as tight as, the UB.

In particular, as we have done in Section 2, we will start by supposing that we are just interested in upper bounding the generalization error of the empirical risk minimizer. Nevertheless, as pointed out in Section 2, DDWUB can be easily generalized also to the lower bounds, or to both lower and upper bounds, and to the general supervised setting with bounded loss functions but, in this work, we did not report all these extensions in order not to make the notation and the presentation over-complicated.

As noted in the introduction, the weights should not depend on the data, but they can depend on some parameters of the data generating distribution. For this reason, we define a set of functions $f_{i}:R^{m}\to R$ with i∈I such that

$$\begin{array}{c} f_{i}\left(R_{1},\cdots ,R_{m}\right)\in \left(0,1\right)\;\forall i\in I,\;\sum_{i\in I}f_{i}\left(R_{1},\cdots ,R_{m}\right)=1. \end{array}$$

In this setting DDWUB can be formulated as follows.

**Corollary** **2.** *If $\forall r_{1},\cdots ,r_{m}\in \left[0,1\right]$*

$$\begin{array}{c} f_{i}\left(r_{1},\cdots ,r_{m}\right)\in \left(0,1\right)\;\forall i\in I,\;\sum_{i\in I}f_{i}\left(r_{1},\cdots ,r_{m}\right)=1, \end{array}$$

*then the following bound holds*

$$\begin{array}{c} P\left\{L\left({\hat{R}}_{i},\frac{\delta }{2m}\right)\leq R_{i}\leq U\left({\hat{R}}_{i},\frac{\delta f_{i}\left(R_{1},\cdots ,R_{m}\right)}{2}\right)\;\forall i\in I\right\}\geq 1-\delta . \end{array}$$

Corollary 2 is a direct consequence of Theorem 2.

To prove that DDWUB outperforms UB we will require some sufficient conditions that L, U, and $f_{i}$ with i∈I must satisfy. Please note that from Corollary 2, it is possible to derive all the lower bounds of the generalization error of the hypotheses in the class since $L\left({\hat{R}}_{i},\frac{\delta }{2m}\right)$ depends just on known quantities. For what concerns, instead, the upper bounds, the answer is not as easy.

In the rest of this section, we will show how to find the upper bound of the generalization error of a hypothesis chosen in H based on DDWUB (Corollary 2) and under which conditions these upper bounds are tighter than the one of UB (Theorem 1). For this purpose

- Lemma 5 will show that under certain conditions, the bound of Corollary 2 can be exploited to compute the upper bound of the generalization error of a hypothesis chosen in a class of possible ones based on the observation of a set of data, by solving a complex optimization problem;
- Lemma 6 will show the conditions under which the optimization problem of Lemma 5 can be simplified;
- Lemma 7 will show the conditions under which the solution of the optimization problem of Lemma 6 is unique;
- Theorem 5 will show the conditions under which the upper bound of the generalization error of Lemma 5 found with Lemma 7 is never looser than the one computed with the UB of Theorem 1. These conditions require the knowledge of a data-dependent threshold;
- Lemma 8 shows that it is possible to estimate this threshold of Theorem 5 in a data independent fashion.

Thanks to Corollary 2 we can state the following lemma.

**Lemma** **5.** *Under the same conditions of Corollary 2, if $\forall \hat{r}\in \left[0,1\right]$ and ∀δ∈(0,1)*

$$\begin{array}{c} L\left(\hat{r},\delta \right)\in \left[0,1\right),\;U\left(\hat{r},\delta \right)\in \left(0,1\right] \end{array}$$

*then the following bound holds with probability at least (1−δ) and ∀i∈I*

$$\begin{array}{cc} R_{i}\leq \max_{r_{1},\cdots ,r_{m}}\; & r_{i}\\ s.t.\; & L\left({\hat{R}}_{j},\frac{\delta }{2m}\right)\leq r_{j}\leq U\left({\hat{R}}_{j},\frac{\delta f_{j}\left(r_{1},\cdots ,r_{m}\right)}{2}\right),\;j\in I. \end{array}$$

The solution of the optimization problem of Lemma 5 is not trivial to be found and its properties are not easy to catch.

The following lemma helps us in simplifying the optimization problem of Lemma 5 under a quite natural condition: the upper bound of the generalization error of a hypothesis should decrease if the generalization error of one of the other hypotheses in the class increases.

**Lemma** **6.** *Under the same conditions of Lemma 5, if ∀r^i∈0,1n,⋯,1, $\forall r_{1},\cdots ,r_{m}\in \left[0,1\right]$ and ∀j∈I\i and $\forall r_{j}^{\prime },r_{j}^{\prime \prime }\in \left[0,1\right]$ such that $r_{j}^{\prime }<r_{j}^{\prime \prime }$*

$$\begin{array}{cc} \; & U\left({\hat{r}}_{i},\frac{\delta f_{i}\left(r_{1},\cdots ,r_{j-1},r_{j}^{\prime },r_{j+1},\cdots ,r_{m}\right)}{2}\right)-U\left({\hat{r}}_{i},\frac{\delta f_{i}\left(r_{1},\cdots ,r_{j-1},r_{j}^{\prime \prime },r_{j+1},\cdots ,r_{m}\right)}{2}\right)\geq 0, \end{array}$$

*where i∈I, then the optimization problem of Lemma 5 is equivalent to the following one*

$$\begin{array}{cc} R_{i}\leq \max_{r_{i}}\; & r_{i}\\ s.t.\; & r_{i}\leq U\left({\hat{R}}_{i},\frac{\delta f_{i}\left(r_{1},\cdots ,r_{m}\right)}{2}\right). \end{array}$$

*where $r_{j}=L\left({\hat{R}}_{j},\frac{\delta }{2m}\right)$, with j∈I\i.*

In fact, the hypotheses of the lemma imply that to reach the maximum or $r_{i}$, one must reach the lower bounds of $r_{j}$ with j∈I\i.

Even if the optimization problem of Lemma 6 is much simpler than the one of Lemma 5, the next result further simplifies it under another sufficient condition which ensure the existence and uniqueness of the solution: the upper bound of the generalization error of a hypothesis should not increase too fast it its generalization error decreases.

**Lemma** **7.** *Under the same conditions of Lemma 6, if ∀r^i∈0,1n,⋯,1, $\forall r_{1},\cdots ,r_{m}\in \left[0,1\right]$, and $\forall r_{i}^{\prime },r_{i}^{\prime \prime }\in \left[0,1\right]$ such that $r_{i}^{\prime }<r_{i}^{\prime \prime }$*

$$\begin{array}{c} \frac{U\left({\hat{r}}_{i},\frac{\delta f_{i}\left(r_{1},\cdots ,r_{i-1},r_{i}^{\prime \prime },r_{i+1},\cdots ,r_{m}\right)}{2}\right)-U\left({\hat{r}}_{i},\frac{\delta f_{i}\left(r_{1},\cdots ,r_{i-1},r_{i}^{\prime },r_{i+1},\cdots ,r_{m}\right)}{2}\right)}{r_{i}^{\prime \prime }-r_{i}^{\prime }}<1, \end{array}$$

then the solution $r_{i}^{*}$ of the optimization problem of Lemma 6 exists, it is unique, and it is the fixed point of the following function of $r_{i}$

$$\begin{array}{c} r_{i}=U\left({\hat{R}}_{i},\frac{\delta f_{i}\left(r_{1},\cdots ,r_{m}\right)}{2}\right), \end{array}$$

*where $r_{j}=L\left({\hat{R}}_{j},\frac{\delta }{2m}\right)$ with j∈I\i.*

In fact, the hypothesis of the lemma guarantees the uniqueness of the solution.

Algorithm 1 reports a simple pseudo-code for finding the fixed point of Lemma 7 based on the bisection method.

The next result introduces a parameter, more specifically a threshold, θ and states the condition over θ which states that the DDWUB improves over the UB.

**Theorem** **5.** *Under the same conditions of Lemma 7, let us consider θ∈[0,1] and suppose that $\forall r_{1},\cdots ,r_{m}\in \left[0,1\right]$ and $\forall r_{j}^{\prime },r_{j}^{\prime \prime }\in \left[0,\theta \right]$*

$$\begin{array}{c} f_{j}\left(r_{1},\cdots ,r_{j-1},r_{j}^{\prime },r_{j+1},\cdots ,r_{m}\right)-f_{j}\left(r_{1},\cdots ,r_{j-1},r_{j}^{\prime \prime },r_{j+1},\cdots ,r_{m}\right)=0, \end{array}$$

*with j∈I. Let us also suppose that if $r_{1},\cdots ,r_{m}\in \left[0,\theta \right]$ then ∀j∈I*

$$\begin{array}{c} f_{j}\left(r_{1},\cdots ,r_{m}\right)=\frac{1}{m}. \end{array}$$

*If*

$$\begin{array}{c} \theta \geq U\left({\hat{R}}_{i},\frac{\delta }{2m}\right), \end{array}$$

*and if $\forall r_{1},\cdots ,r_{m}\in \left[0,1\right]$, ∀j∈I\i, $\forall r_{j}^{\prime },r_{j}^{\prime \prime }\in \left(\theta ,1\right]$ such that $r_{j}^{\prime }<r_{j}^{\prime \prime }$*

$$\begin{array}{c} f_{i}\left(r_{1},\cdots ,r_{j-1},r_{j}^{\prime },r_{j+1},\cdots ,r_{m}\right)-f_{i}\left(r_{1},\cdots ,r_{j-1},r_{j}^{\prime \prime },r_{j+1},\cdots ,r_{m}\right)<0 \end{array}$$

*then the following bound holds*

$$\begin{array}{c} r_{i}^{*}\leq U\left({\hat{R}}_{i},\frac{\delta }{2m}\right). \end{array}$$

The proof of Theorem 5 can be found in Appendix B.

Theorem 5 basically states that under particular conditions, the solution of the problem of Corollary 2 is never looser than the one of Theorem 1.

Unfortunately, we cannot set $\theta =U\left({\hat{R}}_{i},\frac{\delta }{2m}\right)$ since this would be a data-dependent choice which will result in a data-dependent weighting strategy. The next lemma addresses this problem.
**Algorithm 1:** Algorithm for finding the fixed point of Lemma 7 based on the bisection method.
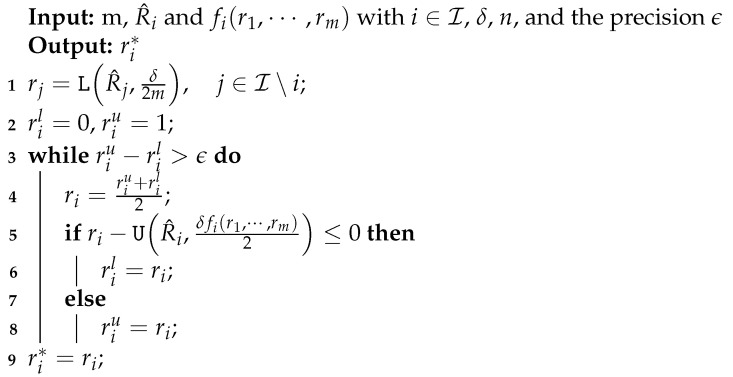

**Lemma** **8.** *Under the same conditions of Theorem 5, if ∀r^,r^′,r^″∈{0,1m,⋯,1} such that ${\hat{r}}^{\prime }<{\hat{r}}^{\prime \prime }$ we have that*

$$\begin{array}{cc} \; & i^{*}=\arg\min_{i\in I}{\hat{R}}_{i},\\ \; & L\left({\hat{r}}^{\prime },\delta \right)-L\left({\hat{r}}^{\prime \prime },\delta \right)\leq 0,\\ \; & \exists L^{-1}:L^{-1}\left(L\left(\hat{r},\delta \right),\delta \right)\geq \hat{r}, \end{array}$$

*then*

$$\begin{array}{c} U\left({\hat{R}}_{i^{*}},\frac{\delta }{2m}\right)\leq U\left(L^{-1}\left(\min\left[R_{1},\cdots ,R_{m}\right],\frac{\delta }{2m}\right),\frac{\delta }{2m}\right). \end{array}$$

The proof of Lemma 8 can be found in Appendix B.

Lemma 8 provides us a method for finding a $\theta \geq U\left({\hat{R}}_{i^{*}},\frac{\delta }{2m}\right)$ in a data independent way by setting

$$\begin{array}{c} \theta =U\left(L^{-1}\left(\min\left[R_{1},\cdots ,R_{m}\right],\frac{\delta }{2m}\right),\frac{\delta }{2m}\right). \end{array}$$

Thanks to all these results we can provide a method for finding the fixed point of Lemma 7 but with data independent weighting strategy which satisfies the hypothesis of Theorem 5 and a data independent θ defined in Lemma 8.

**Lemma** **9.** *Under the same conditions of Lemma 8, Algorithm 2 finds the fixed point of Lemma 7 but with a data independent weighting strategy which satisfies the hypothesis of Theorem 5 and a data independent θ defined in Lemma 8.*

The proof of Lemma 9 can be found in Appendix B.
**Algorithm 2:** Algorithm for finding the fixed point of Lemma 7 but with data independent weighting strategy which satisfies the hypothesis of Theorem 5 and a data independent θ defined in Lemma 8.
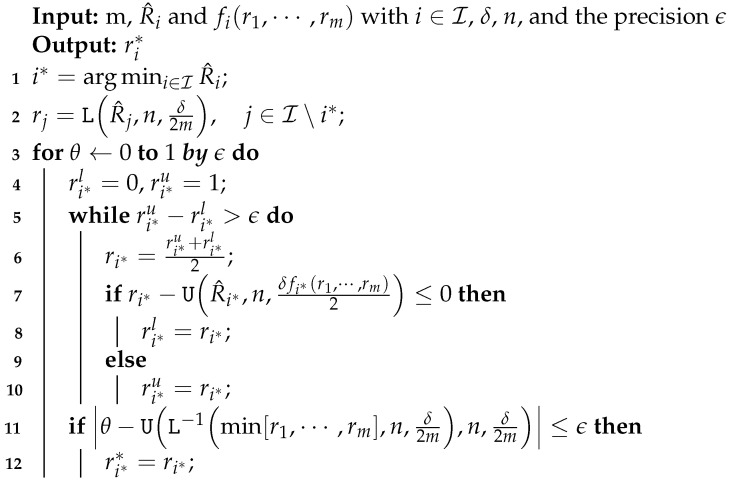


Please note that if we apply this general theory to Corollary 1 we obtain the same results of Section 2.

In the next section, instead, we apply the general theory to a the more complex case of when [15] is employed together with the same weights exploited in Corollary 1.

### 3.1. From Theory to Practice

In this section, we will exploit the same solution of Corollary 1 for the weights needed in DDWUB and we will show that is satisfies hypothesis of the Theorems, the Corollaries, and the Lemmas presented in the previous section. In particular we will set

$$\begin{array}{c} f_{i}\left(R_{1},\cdots ,R_{m}\right)=\frac{e^{-\gamma \max[\theta ,R_{i}]}}{\sum_{j\in I}e^{-\gamma \max[\theta ,R_{j}]}}\;\forall i\in I, \end{array}$$

where γ∈[0,+∞) and θ∈[0,1] are finite constants which regulates the shape of the distribution of the weights.

The following lemma shows that these weighs satisfy the sufficient conditions which states that DDWUB outperforms, or in the worst case performs as, the UB.

**Lemma** **10.** *If γ∈[0,+∞) is a finite constant and*

$$\begin{array}{cc} \; & f_{i}\left(R_{1},\cdots ,R_{m}\right)=\frac{e^{-\gamma \max[\theta ,R_{i}]}}{\sum_{j\in I}e^{-\gamma \max[\theta ,R_{j}]}}\;\forall i\in I, \end{array}$$

*then the hypotheses of Corollary 2 and Theorem 5 are satisfied.*

The proof of Lemma 10 can be found in Appendix B.

Please note that for what concerns the hypothesis of Lemmas 6 and 7, we cannot prove that condition holds without knowing the shape of the lower and upper bounds of the generalization error. For this reason, we exploit the generalization bounds of [15] which are the tightest ones in the settings of this paper [6]. This will allow us in Section 5 to compare the UB with the DDWUB with a set of numerical experiments.

To reach our goal let us define the Regularized Incomplete Beta Function $p=F\left(r;a,b\right)=I_{r}\left(a,b\right)$ and its inverse $r=F^{-1}\left(p;a,b\right)$ with parameters specified by $a\in N^{*}$ and $b\in N^{*}$ for the corresponding values of *r* and probabilities in *p*

$$\begin{array}{c} F\left(r;a,b\right)=\frac{1}{B(a,b)}\int_{0}^{r}t^{a-1}{\left(1-t\right)}^{b-1}dt,\;F^{-1}\left(p;a,b\right)=\left\{r:F(r;a,b)=p\right\}, \end{array}$$

where

$$\begin{array}{c} B\left(a,b\right)=B\left(b,a\right)=\frac{(a-1)!(b-1)!}{(a+b-1)!} \end{array}$$

is the Complete Beta Function.

The following lemma states the conditions under which all the hypotheses of Corollary 2, Lemmas 6 and 7, Theorem 5, and Lemma 8 are satisfied if, in Corollary 2, the lower and upper generalization bounds proposed by [15], recalled by Theorem A2 in the Appendix A, and the weights defined in Lemma 10 are exploited.

**Lemma** **11.** *Let us exploit the lower and upper generalization bounds proposed by [15] in Corollary 2*

$$\begin{array}{cc} \; & L\left(\hat{R},\delta \right)=\left\{\begin{array}{cc} F^{-1}\left(\delta ;n\hat{R},n-n\hat{R}+1\right) & \hat{R}\in \left\{\frac{1}{n},\frac{2}{n},\cdots ,1\right\}\\ 0 & \hat{R}=0 \end{array}\right.,\\ \; & U\left(\hat{R},\delta \right)=\left\{\begin{array}{cc} F^{-1}\left(1-\delta ;n\hat{R}+1,n-n\hat{R}\right) & \hat{R}\in \left\{0,\frac{1}{n},\cdots ,\frac{n-1}{n}\right\}\\ 1 & \hat{R}=1 \end{array}\right., \end{array}$$

*together with the weights defined in Lemma 10. Then if*

$$\begin{array}{cc} \; & i^{*}=\arg\min_{i\in I}{\hat{R}}_{i},\\ \; & {\hat{R}}_{i^{*}}\neq 1,\\ \; & \gamma <\min_{\hat{r}\in \left\{0,\frac{1}{n},\cdots ,\frac{n-1}{n}\right\},\;p\in \left(0,1\right)}\frac{{\left(U\left(\hat{r},\frac{\delta p}{2}\right)\right)}^{n\hat{r}}{\left(1-U\left(\hat{r},\frac{\delta p}{2}\right)\right)}^{n-n\hat{r}-1}}{B\left(n\hat{r}+1,n-n\hat{r}\right)\frac{\delta }{2}p\left(1-p\right)}, \end{array}$$

*the hypotheses of Corollary 2, Lemmas 6 and 7, Theorem 5, and Lemma 8 are satisfied. Moreover, note that*

$$\begin{array}{cc} \; & L^{-1}\left(r,n,\delta \right)=\min\left\{\hat{r}:\hat{r}\in \left\{0,\frac{1}{n},\cdots ,1\right\},r\leq L\left(\hat{r},\delta \right)\right\},\\ \; & \min_{\hat{r}\in \left\{0,\frac{1}{n},\cdots ,\frac{n-1}{n}\right\},\;p\in \left(0,1\right)}\frac{{\left(U\left(\hat{r},\frac{\delta p}{2}\right)\right)}^{n\hat{r}}{\left(1-U\left(\hat{r},\frac{\delta p}{2}\right)\right)}^{n-n\hat{r}-1}}{B\left(n\hat{r}+1,n-n\hat{r}\right)\frac{\delta }{2}p\left(1-p\right)}\geq \frac{2\sqrt{n}}{\sqrt{2\pi }e^{\frac{7}{6}}}. \end{array}$$

The proof of Lemma 11 can be found in Appendix B.

The case where ${\hat{R}}_{i^{*}}=1$ is trivial since, in this case, we can safely state that $R_{i^{*}}\leq 1$.

Please note that the limit in the value of γ is $O(\sqrt{n})$ and this result is connected and in agreement with the consistency results derived in the PAC-Bayes [30] and in Algorithmic Stability [31] theories where the distribution-dependent prior of [27] is exploited.

### 3.2. Observation

Let us consider the case where the empirical errors of the hypotheses in the space have been sorted as follows

$$\begin{array}{c} {\hat{R}}_{1}\leq {\hat{R}}_{2}\leq \cdots \leq {\hat{R}}_{m}, \end{array}$$

and let us exploit the results of Section 3.1.

Let us set

$$\begin{array}{c} \gamma =\frac{\sqrt{n}}{4}<\frac{2\sqrt{n}}{\sqrt{2\pi }e^{\frac{7}{6}}}, \end{array}$$

and suppose that

$$\begin{array}{c} {\hat{R}}_{1}\neq 1. \end{array}$$

Then by using the UB (see Theorem 1) we can state that with probability at least (1−δ)

$$\begin{array}{c} R_{1}\in \left[0,U\left({\hat{R}}_{1},\frac{\delta }{2m}\right)\right], \end{array}$$

while, by using DDWUB (see Lemma 11), we can state that with probability at least (1−δ)

$$\begin{array}{cc} 0\leq R_{1}\leq r_{1}^{*}=\max_{r_{1}}\; & r_{1}\\ s.t.\; & \left\{\begin{array}{c} r_{1}=U\left({\hat{R}}_{1},\frac{\delta f_{1}\left(r_{1},\cdots ,r_{m}\right)}{2}\right)\\ r_{i}=L\left({\hat{R}}_{i},\frac{\delta }{2m}\right),\;i\in I\backslash 1\\ f_{i}\left(r_{1},\cdots ,r_{m}\right)=\frac{e^{-\gamma \max[\theta ,r_{i}]}}{\sum_{j=1}^{m}e^{-\gamma \max[\theta ,r_{j}]}},\;i\in I\\ \gamma =\frac{\sqrt{n}}{4}\\ \theta =U\left(L^{-1}\left(\min\left[r_{1},\cdots ,r_{m}\right],\frac{\delta }{2m}\right),\frac{\delta }{2m}\right) \end{array}\right.. \end{array}$$

By looking at this last problem and by setting $r_{1}=r_{1}^{*}$ for simplicity, we can observe some properties. The first one is that

$$\begin{array}{c} f_{i}\left(r_{1},\cdots ,r_{m}\right)=\left\{\begin{array}{cc} \frac{e^{-\gamma \theta }}{\left|J\right|e^{-\gamma \theta }+\sum_{j=I\backslash J}e^{-\gamma r_{j}}} & i\in J\\ \frac{e^{-\gamma r_{i}}}{\left|J\right|e^{-\gamma \theta }+\sum_{j=I\backslash J}e^{-\gamma r_{j}}} & i\in I\backslash J \end{array}\right.,\;J=\left\{j:j\in I,r_{j}\leq \theta \right\}. \end{array}$$

The second one is that ∀i∈I\J

$$\begin{array}{cc} \frac{e^{-\gamma r_{i}}}{\left|J\right|e^{-\gamma \theta }+\sum_{j=I\backslash J}e^{-\gamma r_{j}}} & \leq \frac{e^{-\gamma \theta }}{\left|J\right|e^{-\gamma \theta }+\sum_{j=I\backslash J}e^{-\gamma r_{j}}}. \end{array}$$

These properties state that DDWUB, with respect to the UB, can discard, or more properly reduce, the risk of the hypotheses $h_{i}$ with i∈I\J when estimating the generalization error of the hypothesis $h_{1}$. As a first raw approximation, we can state that we must pay a risk only for the hypotheses $h_{i}$ with i∈J obtaining

$$\begin{array}{c} R_{1}\in \left[0,U\left({\hat{R}}_{1},\frac{\delta }{2|J|}\right)\right], \end{array}$$

where |J|≤m. A quite important aspect is then to understand some properties of θ. Let us recall that

$$\begin{array}{c} \theta =U\left(L^{-1}\left(\min\left[r_{1},\cdots ,r_{m}\right],\frac{\delta }{2m}\right),\frac{\delta }{2m}\right), \end{array}$$

but we can easily state that

$$\begin{array}{c} \min\left[r_{1},\cdots ,r_{m}\right]=\min\left[U\left({\hat{R}}_{1},\frac{\delta f_{1}\left(r_{1},\cdots ,r_{m}\right)}{2}\right),L\left({\hat{R}}_{2},\frac{\delta }{2m}\right)\right]. \end{array}$$

Thanks to Theorem 5, we can state that $f_{1}\left(r_{1},\cdots ,r_{m}\right)\geq \frac{1}{m}$ and then the case where

$$\begin{array}{c} \min\left[U\left({\hat{R}}_{1},\frac{\delta f_{1}\left(r_{1},\cdots ,r_{m}\right)}{2}\right),L\left({\hat{R}}_{2},\frac{\delta }{2m}\right)\right]=U\left({\hat{R}}_{1},\frac{\delta f_{1}\left(r_{1},\cdots ,r_{m}\right)}{2}\right), \end{array}$$

is quite unusual since it means that ${\hat{R}}_{2}-{\hat{R}}_{1}$ is large, or, in other words, it means that our set of hypotheses contains just one hypothesis with small error and many hypotheses with high error. Instead, if

$$\begin{array}{c} \min\left[U\left({\hat{R}}_{1},\frac{\delta f_{1}\left(r_{1},\cdots ,r_{m}\right)}{2}\right),L\left({\hat{R}}_{2},\frac{\delta }{2m}\right)\right]=L\left({\hat{R}}_{2},\frac{\delta }{2m}\right), \end{array}$$

then

$$\begin{array}{c} \theta =L\left({\hat{R}}_{2},\frac{\delta }{2m}\right), \end{array}$$

which means that the threshold θ is obtained from the upper bound of the second-best hypothesis in the space, namely the hypothesis with the second smallest empirical error. To the best of our knowledge, this result is new since in the past many researchers have tried, with similar approaches but with no supporting theory, in proposing to clean the hypothesis space from the hypotheses with high empirical error. The basic idea of these methods is that it is reasonable to discard all the hypotheses such that the lower bound of their generalization error is greater than $U\left({\hat{R}}_{1},\frac{\delta }{2m}\right)$, namely the upper bound of the generalization error of the hypothesis with the smallest empirical error, see for example [25]. Our theory states, instead, that we can smooth the risk due to the hypotheses such that the lower bound of their generalization error is greater than $U\left({\hat{R}}_{2},n,\frac{\delta }{2m}\right)$, namely the upper bound of the generalization error of the hypothesis with the second smallest empirical error.

## 4. Closed Form Results

In this section, we will report some closed form results regarding the Lemma 11. These examples are useful for providing an idea of the effect of the DDWUB, an extensive comparison with numerical results is provided in Section 5.

Let us consider the case where

$$\begin{array}{c} {\hat{R}}_{1}={\hat{R}}_{2}=0,\;{\hat{R}}_{3}=\cdots ={\hat{R}}_{m}=1. \end{array}$$

Let us set

$$\begin{array}{c} \gamma =\frac{\sqrt{n}}{4}<\frac{2\sqrt{n}}{\sqrt{2\pi }e^{\frac{7}{6}}}, \end{array}$$

and note that

$$\begin{array}{c} \arg\min_{i\in I}{\hat{R}}_{i}=1,\;{\hat{R}}_{1}\neq 1. \end{array}$$

If we use the UB (see Theorem 1) with the [15] we obtain that with probability at least (1−δ) the following bound holds

$$\begin{array}{c} R_{1}\in \left[0,1-\sqrt[{n}]{\frac{\delta }{2m}}\right]. \end{array}$$

If, instead, we use DDWUB (see Lemma 9 and Algorithm 2) we obtain that with probability at least (1−δ) the following bound holds

$$\begin{array}{cc} 0\leq R_{1}\leq r_{1}^{*}=\max_{r}\; & r\\ s.t.\; & \left\{\begin{array}{c} r=1-\sqrt[{n}]{\frac{\delta f_{1}\left(r,r_{2},\cdots ,r_{m}\right)}{2}}\\ f_{1}\left(r,r_{2},\cdots ,r_{m}\right)=\frac{e^{-\gamma \max[\theta ,r]}}{e^{-\gamma \max[\theta ,r]}+\sum_{j=2}^{m}e^{-\gamma \max[\theta ,r_{j}]}}\\ r_{2}=0\\ r_{3}=\cdots =r_{m}=\sqrt[{n}]{\frac{\delta }{2m}}\\ \theta =U\left(L^{-1}\left(\min\left[r,r_{2},\cdots ,r_{m}\right],\frac{\delta }{2m}\right),\frac{\delta }{2m}\right)\\ \gamma =\frac{\sqrt{n}}{4} \end{array}\right.. \end{array}$$

Since we can surely state that

$$\begin{array}{c} \min\left[r_{1}^{*},\cdots ,r_{m}\right]=0, \end{array}$$

then

$$\begin{array}{c} \theta =1-\sqrt[{n}]{\frac{\delta }{2m}}. \end{array}$$

Moreover, thanks to Theorem 5, we can state that

$$\begin{array}{c} r_{1}^{*}\leq \theta . \end{array}$$

Asymptotic Case

Please note that

$$\begin{array}{c} f_{1}\left(r_{1}^{*},r_{2},\cdots ,r_{m}\right)=\frac{1}{2+\left(m-2\right)e^{\frac{\sqrt{n}}{4}\left(1-2\sqrt[{n}]{\frac{\delta }{2m}}\right)}}, \end{array}$$

moreover

$$\begin{array}{c} \lim_{n\to \infty }\frac{1}{2+\left(m-2\right)e^{\frac{\sqrt{n}}{4}\left(1-2\sqrt[{n}]{\frac{\delta }{2m}}\right)}}=\frac{1}{2}. \end{array}$$

Consequently, at least asymptotically, DDWUB can discard entirely the risk due to the hypotheses with high empirical error.

Finite Sample Case

DDWUB obviously gives an advantage, with respect to the UB, until

$$\begin{array}{c} r_{3}=\cdots =r_{m}>\theta , \end{array}$$

This means that we have an advantage in terms of the tightness of the estimated upper bound for $R_{1}$ until

$$\begin{array}{c} \sqrt[{n}]{\frac{\delta }{2m}}>1-\sqrt[{n}]{\frac{\delta }{2m}}, \end{array}$$

and consequently until

$$\begin{array}{c} m<\delta 2^{n-1}. \end{array}$$

## 5. Numerical Results

This section is devoted to the numerical comparison between the UB (see Theorem 1) and the DDWUB (see Lemma 11).

In particular we will focus on upper bounding the generalization error of the hypothesis, in the set of possible ones, characterized by the smallest empirical error. The comparison between the UB and the DDWUB will be made in different scenarios to better understand the advantages of the DDWUB.

Scenario A.

Scenario A is an optimistic scenario, the same of Section 4: the set of hypotheses contains few useful hypotheses (small empirical error) and a lot of useless hypotheses (high empirical error) such that

$$\begin{array}{c} {\hat{R}}_{1}={\hat{R}}_{2}=0,\;{\hat{R}}_{3}=\cdots ={\hat{R}}_{m}=1. \end{array}$$

We set δ=0.05 and set the numerical precision of the algorithms to ϵ=0.0001.

**Figure 1:**
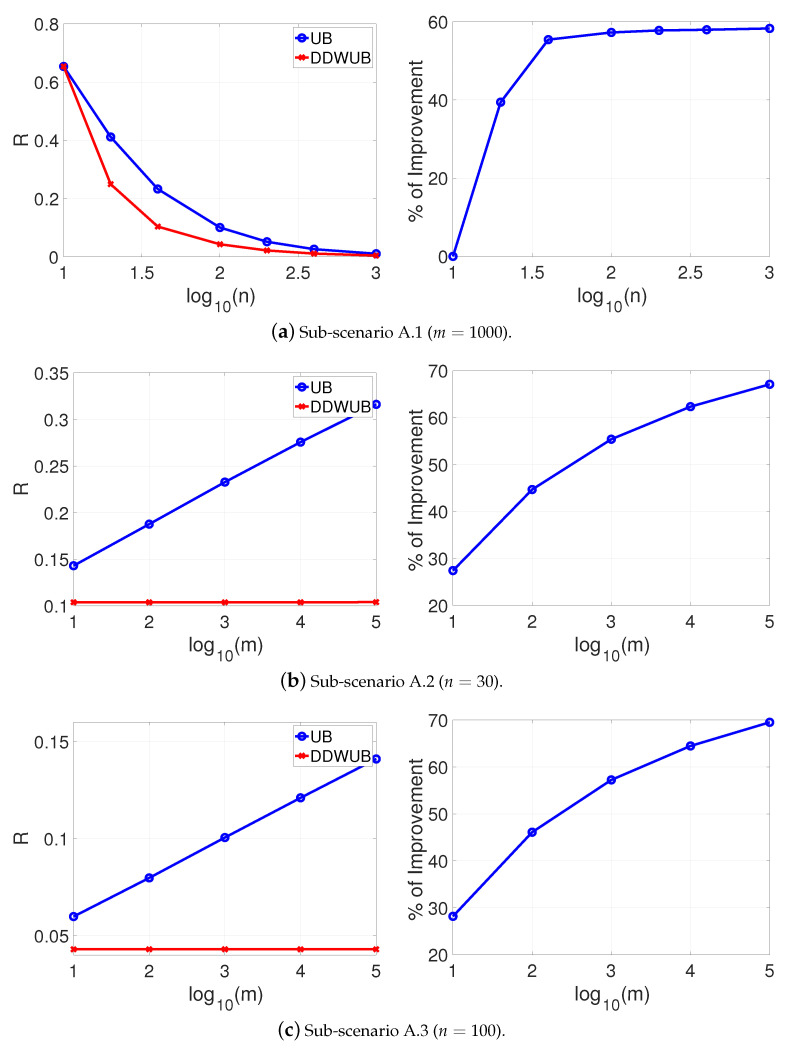
Scenario A (${\hat{R}}_{1}={\hat{R}}_{2}=0$ and ${\hat{R}}_{3}=\cdots ={\hat{R}}_{m}=1$): upper bound of the generalization error of the hypothesis with the smallest empirical error computed with the UB and the DDWUB together with the percentage of improvement.

Figure 1 reports the estimated generalization error upper bound of the hypothesis with the smallest empirical error estimated with the UB and the DDWUB, together with the percentage of improvement in three different sub-scenarios:

- Sub-scenario A.1 (Figure 1a): we set m=1000 and we vary n∈{10,20,40,100,200,400,1000};
- Sub-scenario A.2 (Figure 1b): we vary m∈{10,100,1000,10000,100000} and we set n=40;
- Sub-scenario A.3 (Figure 1c): we vary m∈{10,100,1000,10000,100000} and we set n=100.

Based on the results reported in Figure 1 we can observe that

- DDWUB is always tighter, or equivalent in the worst case, with respect to the UB;
- increasing the number of samples always increases the advantage of the DDWUB over the UB until all the risk of the hypothesis with largest empirical error is disregarded;
- increasing *m* increases the advantage of the DDWUB over the UB until a limit value for *m*: if too many useless hypotheses are present it is not able anymore to disregard their risk. Nevertheless, the larger is *n* the far is this value for *m*.

**Figure 2:**
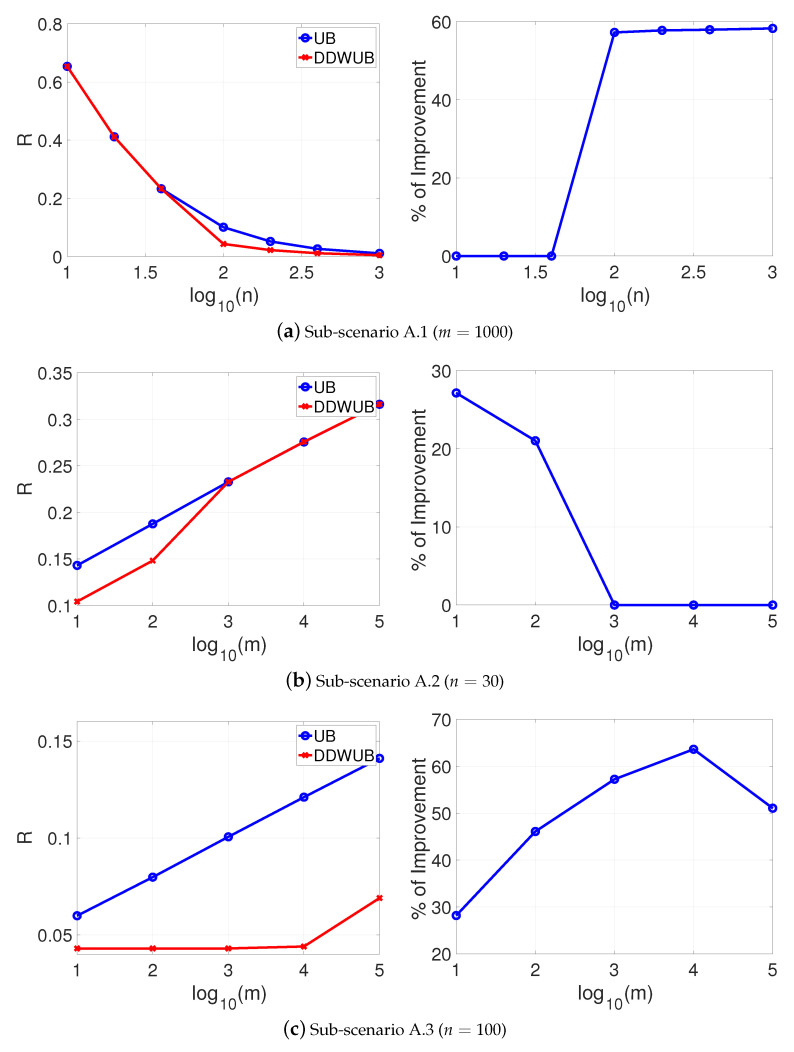
Scenario A (${\hat{R}}_{1}={\hat{R}}_{2}=0$ and ${\hat{R}}_{3}=\cdots ={\hat{R}}_{m}=\frac{1}{2}$): upper bound of the generalization error of the hypothesis with the smallest empirical error computed with the UB and the DDWUB together with the percentage of improvement.

In a slightly less optimistic scenario when

$$\begin{array}{c} {\hat{R}}_{1}={\hat{R}}_{2}=0,\;{\hat{R}}_{3}=\cdots ={\hat{R}}_{m}=\frac{1}{2}, \end{array}$$

which is the case of lot of charlatans and just a few good candidates in a hiring process [34], the results are reported in Figure 2a–c and do not change too much, apart from the limit of *m* when the DDWUB stops to improve over the UB which is obviously smaller.

Scenario B.

The second scenario is a more classical one when

$$\begin{array}{c} {\hat{R}}_{1}=0,\;{\hat{R}}_{2}=\frac{1}{n},\;\cdots ,\;{\hat{R}}_{n+1}=1. \end{array}$$

This case is exploited in many applications (e.g., the CSDB of [20], or the Structural Risk Minimization of [2], or the Structural Risk Minimization over data-dependent hierarchies of [19]).

Also, in this scenario we set δ=0.05 and we set the numerical precision to ϵ=0.0001. Then we vary n∈{10,20,40,100,200,400,1000}.

**Figure 3:**
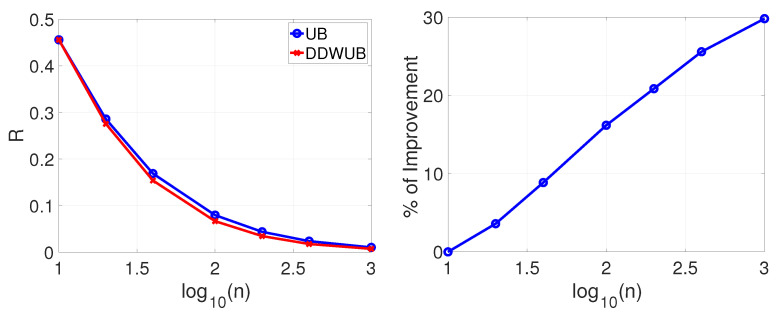
Scenario B: upper bound of the generalization error of the hypothesis with the smallest empirical error computed with the UB and the DDWUB together with the percentage of improvement.

Figure 3 reports the estimated generalization error upper bound of the hypothesis with the smallest empirical error estimated with the UB and the DDWUB, together with the percentage of improvement.

Figure 3 clearly shows the advantage of the DDWUB over the UB and the improvement in the advantage as soon as *n* increases.

Scenario C.

The last scenario involves the unlucky case in which our set of *m* hypotheses is taken from all $2^{n}$ possible binary hypotheses over *n* data. Please note that the number of hypotheses with *i* errors in the $2^{n}$ possible binary hypotheses over *n* data are ni. Then we force our *m* hypotheses to have at least one hypothesis for each possible value of the empirical error and consequently m≥n+1. The remaining m−n−1 are taken from the $2^{n}$ possible binary hypotheses over *n* to approximate the distribution of the $2^{n}$ possible binary hypotheses. The result of this approach is that our set of hypotheses will be composed by m=∑j=0nnj2nz hypotheses, with $z\in N^{*}$, as follows

R^1=⋯=R^n02nz=0,R^n02nz+1=⋯=R^n02nz+n12nz=1n,⋯R^∑j=0n−1nj2nz+1=⋯=R^∑j=0nnj2nz=1.

Please note that for example, when z=1 we obtain the Scenario B.

Also, in this scenario we set δ=0.05 and we set the numerical precision to ϵ=0.0001.

**Figure 4:**
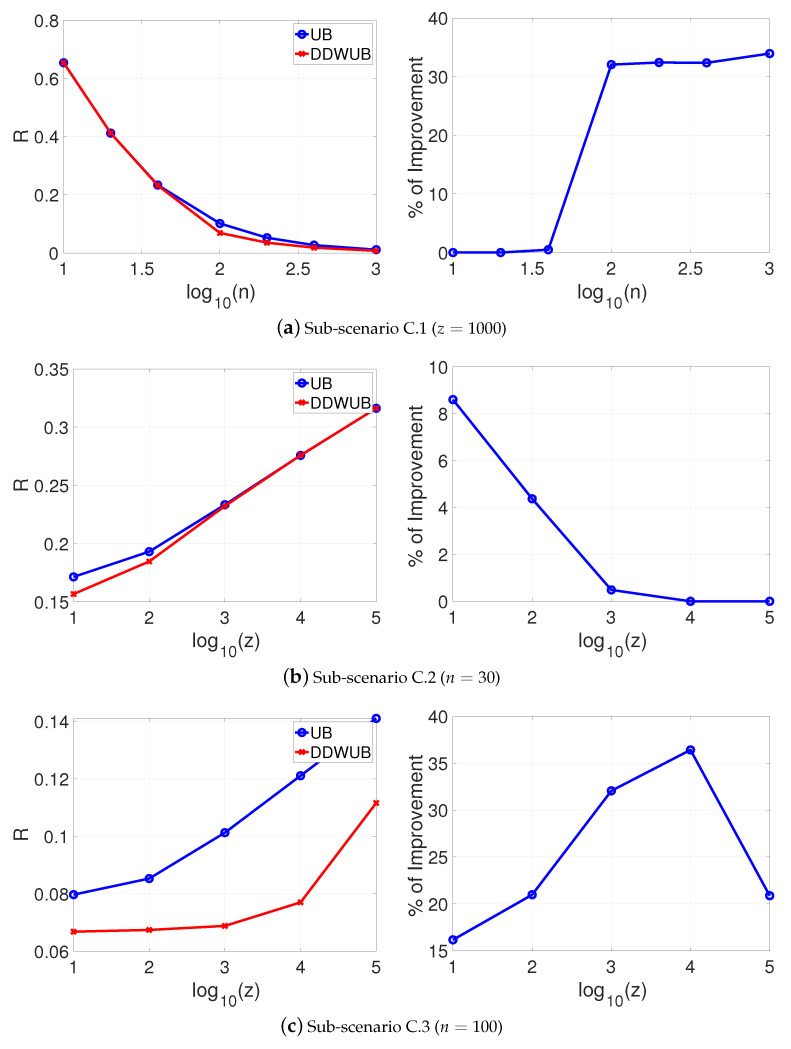
Scenario C: upper bound of the generalization error of the hypothesis with the smallest empirical error computed with the UB and the DDWUB together with the percentage of improvement.

Figure 4a–c reports the estimated generalization error upper bound of the hypothesis with the smallest empirical error estimated with the UB and the DDWUB, together with the percentage of improvement in the same sub-scenarios of Scenario A.

Figure 4 shows that even in this unlucky case, the DDWUB can remarkably outperform the UB.

### The Importance of γ and θ

In this section, we would like to discuss the importance of γ and θ also by means of some numerical experiments supporting our claims.

**Figure 5:**
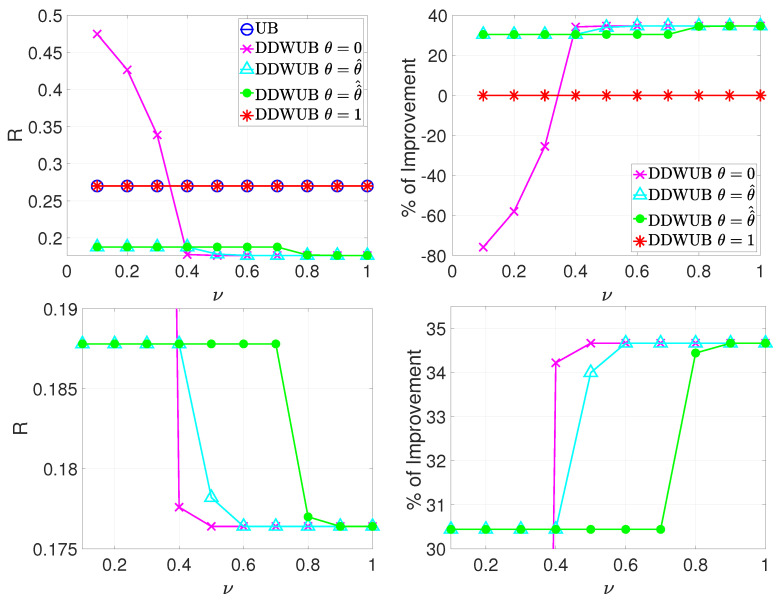
Scenario A (${\hat{R}}_{1}=0.1$, ${\hat{R}}_{2}=\nu$ and ${\hat{R}}_{3}=\cdots ={\hat{R}}_{m}=1$): upper bound of the generalization error of the hypothesis with the smallest empirical error computed with the UB and the DDWUB with $\theta \in \{0,\hat{\theta },\hat{\hat{\theta }},1\}$ together with the percentage of improvement when we set δ=0.05, n=100, and m=1000 and we vary ν∈{0.1,0.2,⋯,1}. The two figures above depict the whole range while the two figures below report a zoom on the most interesting parts.

Let us start with θ. From one side setting θ=1 would make the DDWUB degenerate in the UB eliminating all the benefits of using the DDWUB. From the other side setting θ=0, namely removing θ, is not possible because of the constraint on θ of Theorem 5 which does not allow us, in this case, to guarantee that the solution of the DDWUB always outperform, or in the worst case degenerates in, the UB. Hence, θ∈(0,1) deals with the fact that we do not know the generalization error of our hypotheses, then we must estimate it, and consequently hypotheses with a small but close empirical error cannot be distinguished. Unfortunately, the constraint of Theorem 5 on θ is data-dependent and consequently we must resort to a suboptimal, but data independent, limitation on θ as reported in Lemma 8. The question which raises here is the practical difference of all these choices. For this reason, we consider the Scenario A previously defined with ${\hat{R}}_{1}=0.1$, ${\hat{R}}_{2}=\nu$ and ${\hat{R}}_{3}=\cdots ={\hat{R}}_{m}=1$, where we set δ=0.05, n=100, and m=1000. Then we vary ν∈{0.1,0.2,⋯,1}, and we reported in Figure 5 the comparison between the UB and the DDWUB with θ=0, $\theta =\hat{\theta }$ i.e., equal to the lower limit of the constraint of Theorem 5, $\theta =\hat{\hat{\theta }}$ i.e., equal to the lower limit of the constraint of Lemma 8, and θ=1.

From Figure 5 it is possible to derive some observations. Setting θ=0 in the DDWUB can result in worse estimates with respect to the ones of the UB since when ${\hat{R}}_{2}$ is close to ${\hat{R}}_{1}$ (small ν) we cannot distinguish between the first two hypotheses which are the ones with the lowest generalization error. As soon as ν grows the difference between ${\hat{R}}_{1}$ and ${\hat{R}}_{2}$ becomes statistical relevant and so setting θ=0 in the DDWUB results in better estimates with respect to the UB or even better that the ones of the DDWUB since θ in this particular scenario for large ν is useless. Setting $\theta =\hat{\theta }$ gives the best results and always outperform the UB while setting $\theta =\hat{\hat{\theta }}$ results in worse estimates but still better than the ones of the UB. Please note that for small and large ν, $\hat{\theta }$ and $\hat{\hat{\theta }}$ are equivalent while there is a middle range of ν for which $\hat{\theta }$ performs better $\hat{\hat{\theta }}$. The explanation of this phenomena can be derived using the observations of Section 3.2. The hypothesis that regulates $\hat{\theta }$ is the one corresponding to ${\hat{R}}_{1}$ (the one with the smallest empirical error). The hypothesis that regulates $\hat{\hat{\theta }}$, instead, is the one corresponding to ${\hat{R}}_{2}$ (the second-best hypothesis) if the distance between ${\hat{R}}_{1}$ and ${\hat{R}}_{2}$ is small, i.e., for small ν, while is the one corresponding to ${\hat{R}}_{1}$ if the distance between ${\hat{R}}_{1}$ and ${\hat{R}}_{2}$ is large. Consequently, when ${\hat{R}}_{1}$ and ${\hat{R}}_{2}$ are close it is indifferent to choose one or the other and the estimates of the DDWUB are almost equivalent. When, instead, the difference between ${\hat{R}}_{1}$ and ${\hat{R}}_{2}$ increases, ${\hat{R}}_{2}$, instead of ${\hat{R}}_{1}$, regulates θ and the DDWUB with $\hat{\theta }$ performs better than the DDWUB with $\hat{\hat{\theta }}$. Finally, when the distance between ${\hat{R}}_{1}$ and ${\hat{R}}_{2}$ is large, θ is regulated by ${\hat{R}}_{1}$ both for $\hat{\theta }$ and $\hat{\hat{\theta }}$ and consequently the corresponding estimates of the DDWUB are equivalent. Finally, setting θ=1 results in the UB.

**Figure 6:**
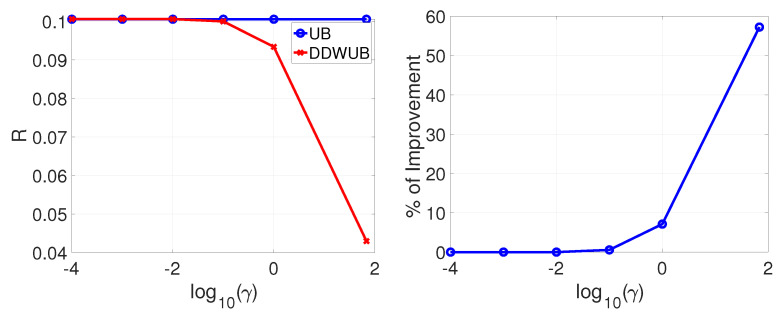
Scenario A (${\hat{R}}_{1}={\hat{R}}_{2}=0$ and ${\hat{R}}_{3}=\cdots ={\hat{R}}_{m}=1$): upper bound of the generalization error of the hypothesis with the smallest empirical error computed with the UB and the DDWUB together with the percentage of improvement when we set n=100 and m=1000 and we vary $\gamma \in \{10^{-5}\sqrt{n},10^{-4}\sqrt{n},\cdots ,10^{-1}\sqrt{n},\hat{\gamma }\}$, where $\hat{\gamma }$ is the limit defined in Lemma 11.

Let us now consider γ. From one side setting γ=0 would make the DDWUB degenerate in the UB eliminating all the benefits of using the DDWUB. From the other side setting γ=∞ would result in splitting equally the confidence just over the hypotheses with estimated error less than θ, not considering all the other hypotheses, which is our scope but unfortunately this is not possible because of the constraints of Lemma 7, which then result in the limitation over γ in Lemma 11. This is the reason we set, in the experiments, γ to the limits of what Lemma 11 allows. After that limit we do not know how the solution of the DDWUB behaves since we do not even know how to retrieve it. Setting a γ smaller than the maximum value allowed by Lemma 11 would diminish the performance of the DDWUB until the DDWUB degenerates in the UB. To support this statement we consider Scenario A with ${\hat{R}}_{1}={\hat{R}}_{2}=0$ and ${\hat{R}}_{3}=\cdots ={\hat{R}}_{m}=1$, then set δ=0.05, n=100 and m=1000, and we vary $\gamma \in \{10^{-5}\sqrt{n},10^{-4}\sqrt{n},\cdots ,10^{-1}\sqrt{n},\hat{\gamma }\}$, where $\hat{\gamma }$ is the limit defined in Lemma 11, and we reported the comparison between the DDWUB and the UB in Figure 6.

From Figure 6 it is possible to clearly observe that the maximum improvement is achieved when γ is maximum and consequently when $\gamma =\hat{\gamma }$. The same result can be observed in all the other scenarios.

## 6. What About the Computable Shell Decomposition Bounds?

In this section, we will show that the DDWUB also improves over the CSDB. Before starting the comparison, we must recall this milestone result.

**Theorem** **6** ([20]). *The following bounds hold*

$$\begin{array}{c} P\left\{R_{i}\leq \max\left\{r:r\in \left[0,1\right],\mathrm{kl}({\hat{R}}_{i}\left||r\right)\leq \frac{\hat{S}\left(\left\lceil \left\lceil r\right\rceil \right\rceil ,n,\delta \right)+\ln\left(\frac{4n}{\delta }\right)}{n}\right\}\;\forall i\in I\right\}\geq 1-\delta , \end{array}$$

*where ⌈⌈r⌉⌉=max[1,⌈rn⌉]n∈{1n,⋯,nn} and if ⌈⌈r⌉⌉=kn then r∈[k−1n,km], kl(q||p)=qlnqp+(1−q)ln1−q1−p is the Kullback–Leibler divergence, and*

$$\begin{array}{c} \hat{S}\left(\frac{k}{n},n,\delta \right)=\ln\left(\max\left[1,2\left|\left\{h_{i}:i\in I,\left|{\hat{R}}_{i}-\frac{k}{n}\right|\leq \frac{1}{n}+\sqrt{\frac{\ln\left(\frac{16n^{2}}{\delta }\right)}{2n-1}}\right\}\right|\right]\right). \end{array}$$

Let us consider the same scenario of Section 4.

The DDWUB is able, at least asymptotically, to discard all risk associated with the hypotheses with high empirical error obtaining that for *n* large enough, the rate of convergence of the bound on $R_{1}$ is O(1n).

Instead, if we use the CSDB of Theorem 6 we get that for *n* large enough, the rate of convergence of the bound on $R_{1}$ is O(ln(n)n). Moreover, note that O(ln(n)n) is also the fastest possible rate of convergence for Theorem 6.

**Figure 7:**
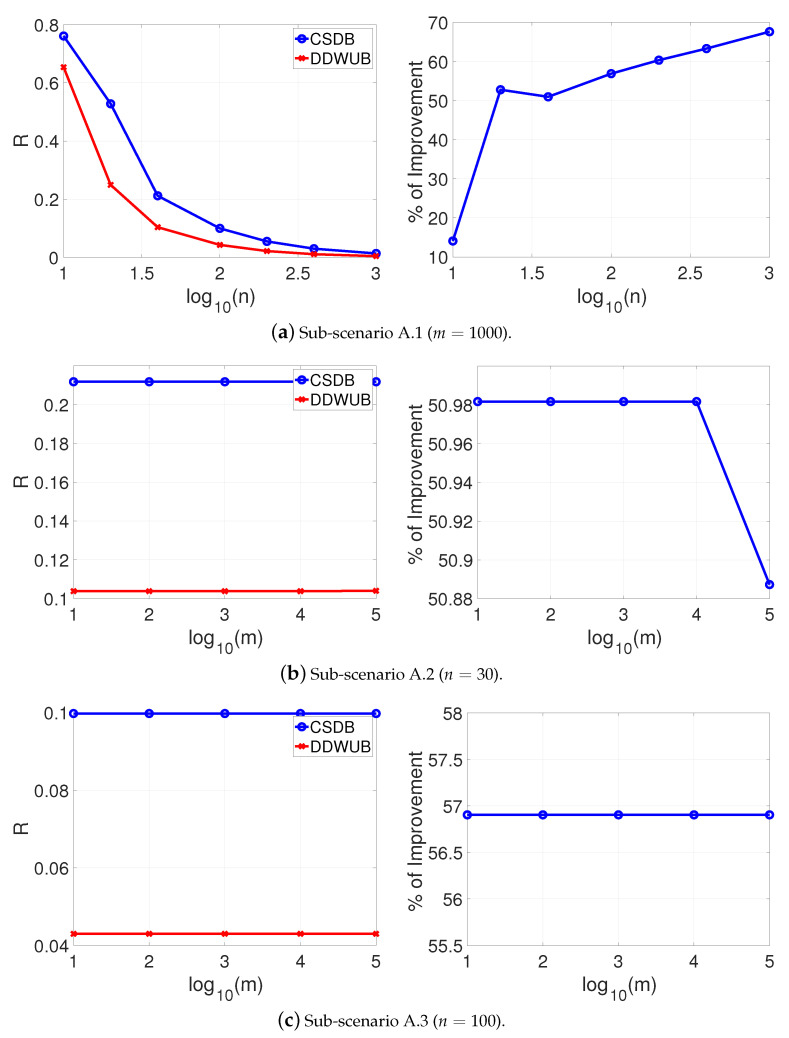
Scenario A (${\hat{R}}_{1}={\hat{R}}_{2}=0$ and ${\hat{R}}_{3}=\cdots ={\hat{R}}_{m}=1$): upper bound of the generalization error of the hypothesis with the smallest empirical error computed with the CSDB and the DDWUB together with the percentage of improvement.

**Figure 8:**
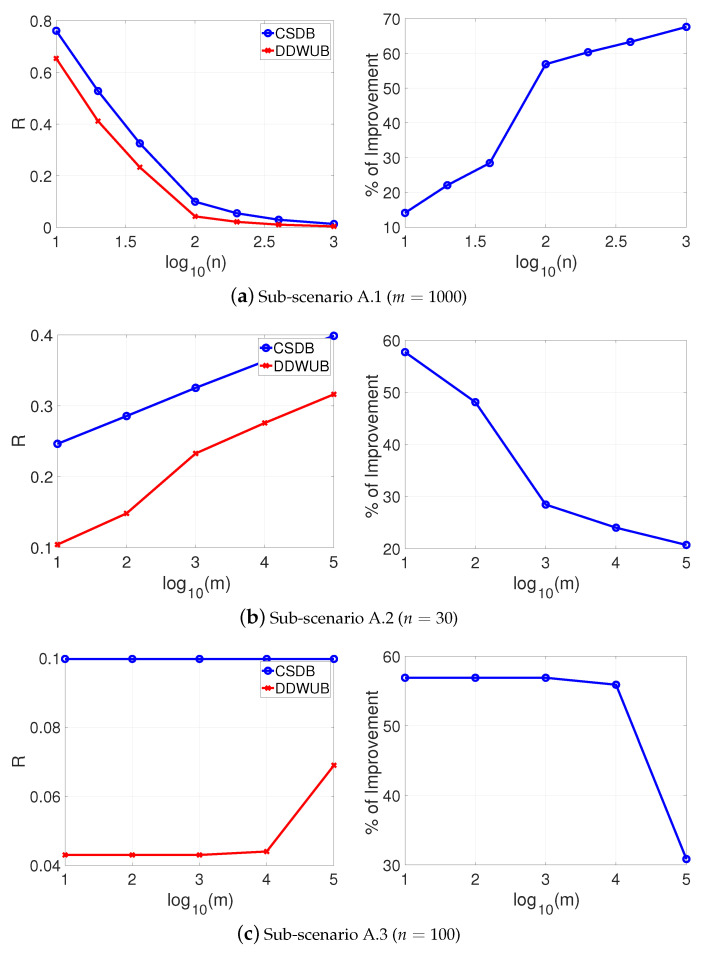
Scenario A (${\hat{R}}_{1}={\hat{R}}_{2}=0$ and ${\hat{R}}_{3}=\cdots ={\hat{R}}_{m}=\frac{1}{2}$): upper bound of the generalization error of the hypothesis with the smallest empirical error computed with the CSDB and the DDWUB together with the percentage of improvement.

**Figure 9:**
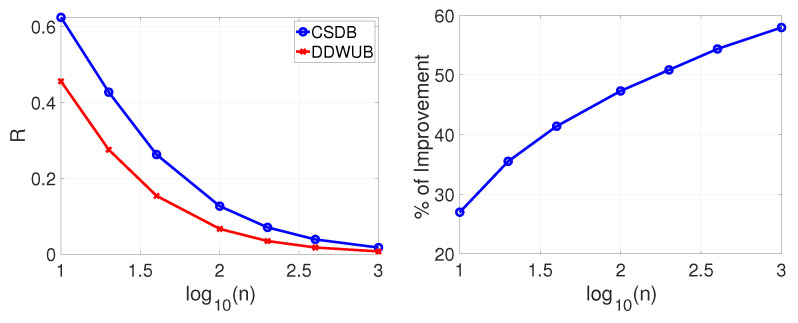
Scenario B: upper bound of the generalization error of the hypothesis with the smallest empirical error computed with the CSDB and the DDWUB together with the percentage of improvement.

**Figure 10:**
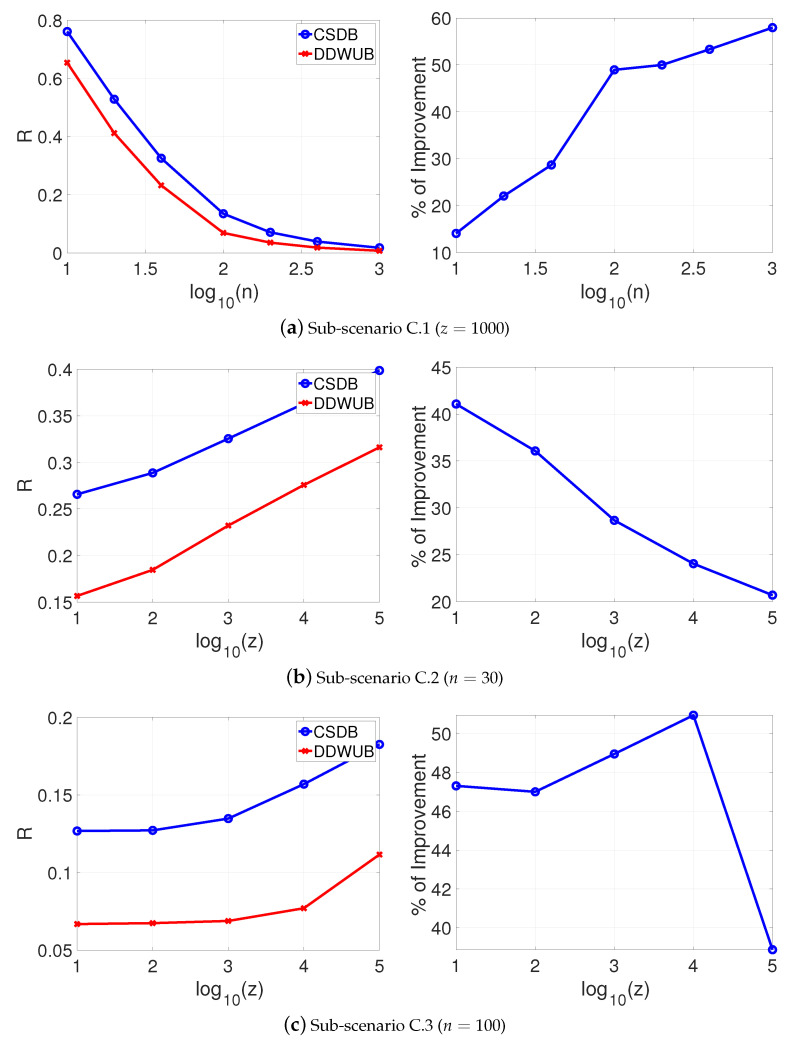
Scenario C: upper bound of the generalization error of the hypothesis with the smallest empirical error computed with the CSDB and the DDWUB together with the percentage of improvement.

If instead of checking the asymptotic behavior of the DDWUB and the CSDB we check their finite sample behavior by means of numerical experiments as in Section 5, we can derive other interesting observations. Figure 7, Figure 8, Figure 9 and Figure 10 (and the associated sub-figures) report the same comparison of Figure 1, Figure 2, Figure 3 and Figure 4 in Section 5 but, instead of comparing the UB with the DDWUB, here we compare the CSDB with the DDWUB.

As it can be clearly seen from the results the DDWUB performs consistently better than the CSDB.

## 7. Improving the Computable Shell Decomposition Bounds

The purpose of this section is to demonstrate that the DDWUB can be exploited to improve known results in Statistical Learning Theory. In particular, this section we will show that DDWUB can be exploited to improve the CSDB.

The proof of the CSDB of Theorem 6 relies on two main results, reported in the next theorem, combined with the UB.

**Theorem** **7** ([20]). *The following bounds hold*

$$\begin{array}{cc} \; & P\left\{\mathrm{kl}({\hat{R}}_{i}\left|\right|R_{i})\leq \frac{\ln\left(|H|\right)+\ln\left(\frac{2}{\delta }\right)}{n}\;\forall i\in I\right\}\geq 1-\delta ,\\ \; & P\left\{S\left(\frac{k}{n},n\right)\leq \hat{S}\left(\frac{k}{n},n,2n\delta \right)\right\}\geq 1-\delta , \end{array}$$

*where*

$$\begin{array}{c} S\left(\frac{k}{n},n\right)=\ln\left(\max\left[1,2\left|\left\{h_{i}:i\in I,R_{i}\in \left[\frac{k-1}{n},\frac{k}{n}\right]\right\}\right|\right]\right) \end{array}$$

*and $\hat{S}\left(\frac{k}{n},n,\delta \right)$ is defined as in Theorem 6.*

By splitting the hypotheses in shells based on their generalization error, namely Hr=hi:i∈I,Ri∈k−1n,kn with r∈{1n,2n,⋯,1}, by combining the two probabilistic bounds of Theorem 7, by using the UB, and by considering the worst case scenario, the result of Theorem 6 is derived. Consequently, [20] consider 2n probabilistic bounds, two for each of one the shells, and spread the confidence (risk) equally over them.

We propose, instead, to use the same approach of the DDWUB in the CSDB. Instead of spreading the confidence equally over the 2n probabilistic bounds, we spread the confidence over them based on the maximum generalization error of the function in each of the *n* shells to which the bounds refer. The results is reported in the following lemma.

**Lemma** **12.** *The following bound holds*

$$\begin{array}{c} P\left\{R_{i}\leq \max\;\left\{\;r:r\in \left[0,1\right],\mathrm{kl}({\hat{R}}_{i}||r)\leq \frac{\hat{S}\left(\left\lceil \left\lceil r\right\rceil \right\rceil ,n,np\left(\left\lceil \left\lceil r\right\rceil \right\rceil \right)\delta \right)+\ln\left(\frac{4}{\delta p(\lceil \lceil r\rceil \rceil )}\right)}{n}\;\right\}\;\;\forall i\in I\;\right\}\;\geq 1-\delta , \end{array}$$

*where*

$$\begin{array}{c} p\left(r\right)=\frac{e^{-\frac{nr}{\ln(n)}}}{\sum_{i=1}^{n}e^{-\frac{i}{\ln(n)}}}. \end{array}$$

The proof is the simple application of the concepts behind the DDWUB. θ is not needed since for each one of the shells we exactly know by definition the maximum generalization error of the functions inside it. γ is set to nln(n) and the reason is the following one. The rate of convergence of CSDB is O(ln(n)n) in the general case and O(ln(n)n) when ${\hat{R}}_{i}=0$. Let us study instead the rate of convergence of the bound of Lemma 12. Thanks to the Geometric Series we can state that

$$\begin{array}{c} p\left(r\right)=\frac{e^{-\frac{nr}{\ln(n)}}}{\sum_{i=1}^{n}e^{-\frac{i}{\ln(n)}}}=e^{-\frac{nr}{\ln(n)}}\frac{1-e^{\frac{1}{\ln(n)}}}{1-e^{\frac{n}{\ln(n)}}} \end{array}$$

For *n* large enough we can state that

$$\begin{array}{c} \ln\left(\frac{1}{p(r)}\right)\approx \frac{nr}{\ln(n)}+\ln\left(\ln\left(n\right)\right) \end{array}$$

Consequently, the rate of convergence of the bound of Lemma 12 is O(ln(ln(n))n) in the general case and O(ln(ln(n))n) when ${\hat{R}}_{i}=0$. This means that for γ=nln(n) the rate of convergence of the bound of Lemma 12 of better than the one of CSDB.

It could be possible to improve the bound with a different values of γ and θ or to prove that for particular values of γ and θ, Lemma 12 is always better than the CSDB but this is beyond of the scope in this paper.

**Figure 11:**
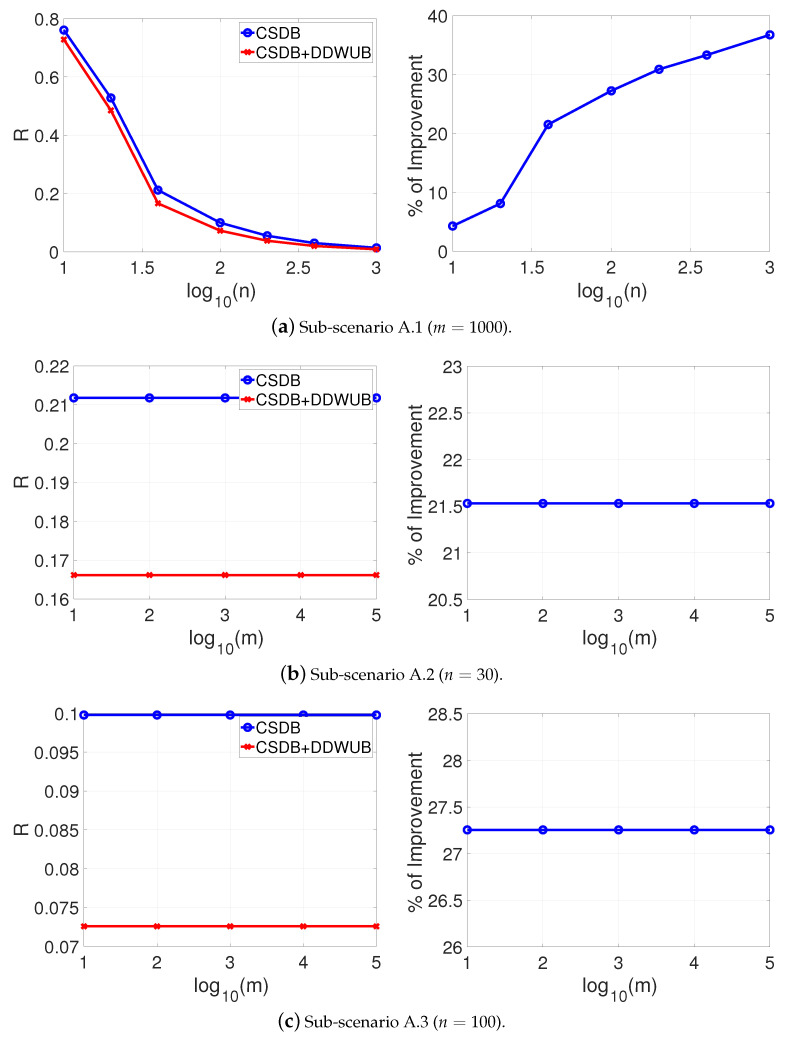
Scenario A (${\hat{R}}_{1}={\hat{R}}_{2}=0$ and ${\hat{R}}_{3}=\cdots ={\hat{R}}_{m}=1$): upper bound of the generalization error of the hypothesis with the smallest empirical error computed with the CSDB and the CSDB+DDWUB together with the percentage of improvement.

**Figure 12:**
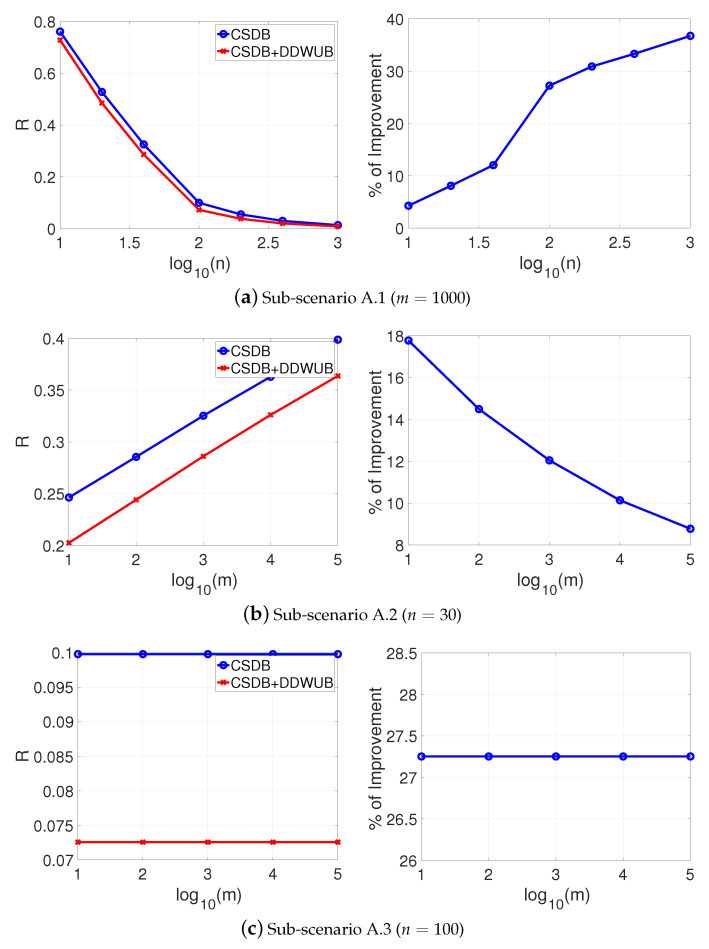
Scenario A (${\hat{R}}_{1}={\hat{R}}_{2}=0$ and ${\hat{R}}_{3}=\cdots ={\hat{R}}_{m}=\frac{1}{2}$): upper bound of the generalization error of the hypothesis with the smallest empirical error computed with the CSDB and the CSDB+DDWUB together with the percentage of improvement.

**Figure 13:**
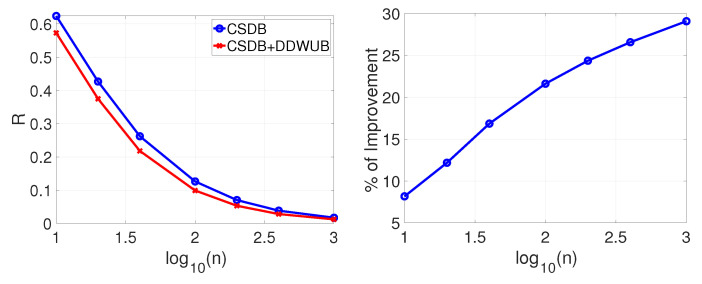
Scenario B: upper bound of the generalization error of the hypothesis with the smallest empirical error computed with the CSDB and the CSDB+DDWUB together with the percentage of improvement.

**Figure 14:**
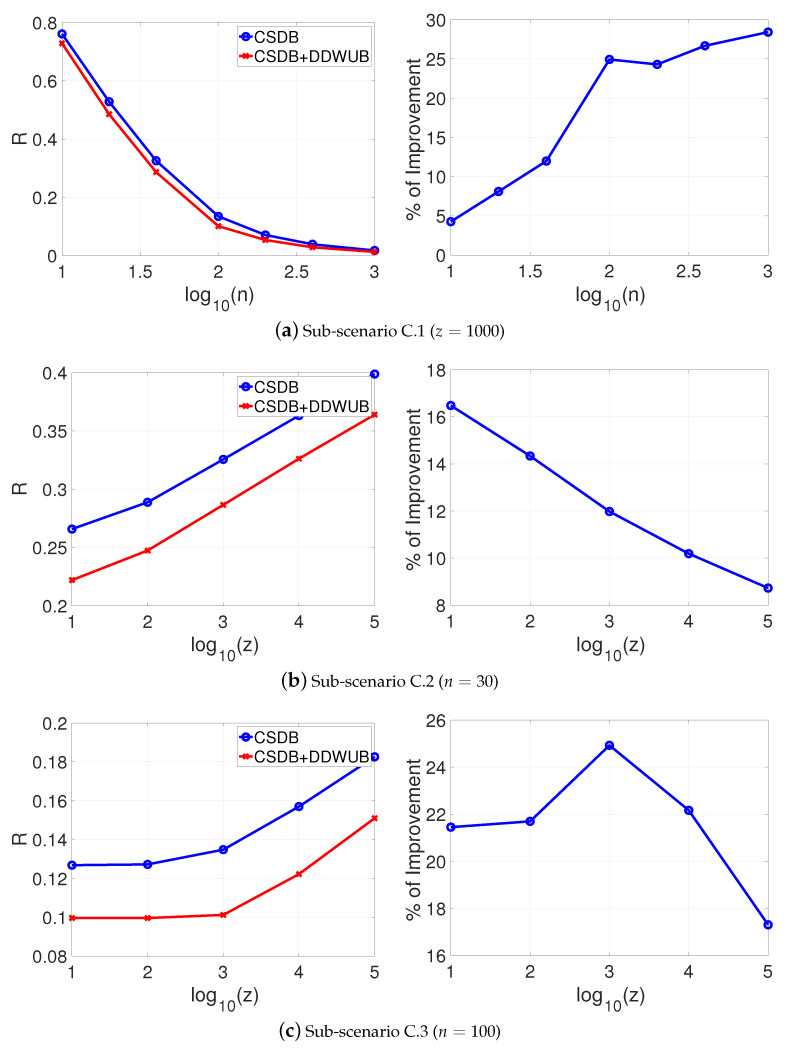
Scenario C: upper bound of the generalization error of the hypothesis with the smallest empirical error computed with the CSDB and the CSDB+DDWUB together with the percentage of improvement.

If, instead, we compare the finite sample behavior of the CSDB and the Lemma 12 (that we will call CSDB+DDWUB) by means of numerical experiments as in Section 5, we can observe the possible benefit of using DDWUB in CSDB, a well-known result of Statistical Learning Theory. Figure 11, Figure 12, Figure 13 and Figure 14 (and the associated sub-figures) report the same comparison of Figure 1, Figure 2, Figure 3 and Figure 4 in Section 5 but, instead of comparing the UB with the DDWUB, here we compare the CSDB with the CSDB+DDWUB.

As it can be clearly seen from the results the CSDB+DDWUB performs consistently better than the CSDB.

## 8. Conclusions and Discussion

In this work we derived, for an arbitrary finite hypothesis space, a fully empirical new upper bound on the generalization error of the hypothesis of minimal training error. As noted in the paper, although we presented just the upper bound, our result can be easily generalized also to lower or both upper and lower bounds.

In particular we depicted a quite general framework under which it is possible to improve the Union Bound with a distribution depended weighting strategy associated with the risk of each choice. Then we stated the conditions under which the proposed Distribution-Dependent Weighed Union Bound is always tighter than the one based on the Union Bound. We showed that these conditions are quite easy to satisfy. By means of both closed form and numerical results we demonstrated that Distribution-Dependent Weighed Union Bound is consistently tighter than the Union Bound in different scenarios. Finally, we showed that the Distribution-Dependent Weighed Union Bound is also able to improve over the Computable Shell Decomposition Bound, another quite powerful distribution-dependent Union Bound.

The results of this work are quite promising and pave the way toward many different future improvements. One is surely to derive a class of weighting strategies which satisfies behind the Distribution-Dependent Weighed Union Bound. Another one is to understand how to exploit the results of this work for improving all the results in Statistical Learning Theory where the Union Bound is employed. A final one, and probably the most important one, is how to extend and apply our results to the infinite-dimensional hypothesis spaces. This extension, which is obviously not trivial, has multiple alternatives which can be speculated. The first, and naive, approach would be to plug our approach into the compression bound which already deals with the infinite-dimensional case, exploiting the concept of compression, and exploits naively the union bound. The second approach, less intuitive, would be to split the hypothesis spaces in a finite number of shells, estimate the size of each shell with classical bounds based on the Vapnik–Chervonenkis or Rademacher Complexity theories and then exploit our Distribution-Dependent Weighed Union Bound to pay the price of the choice of one of the shells. The last, and more challenging, approach would be to plug our weighting strategy directly in the derivation of the Vapnik–Chervonenkis or Rademacher Complexity bases bounds shrinking then the measures of complexity as alternative to the localization approaches.

## Data Availability

See Appendix D.

## Appendix A. Known Results

Known bounds and results exploited in the paper are included in this section.

**Theorem** **A1** ([16]). *The following bounds hold*

$$\begin{array}{c} P\left\{R\geq \hat{R}-\sqrt{\frac{\ln\left(\frac{1}{\delta }\right)}{2n}}\right\}\geq 1-\delta ,\;P\left\{R\leq \hat{R}+\sqrt{\frac{\ln\left(\frac{1}{\delta }\right)}{2n}}\right\}\geq 1-\delta . \end{array}$$

**Theorem** **A2** ([15]). *The following bounds hold*

$$\begin{array}{cc} \; & P\left\{R\geq \left\{\begin{array}{cc} F^{-1}\left(\delta ;n\hat{R},n-n\hat{R}+1\right) & \hat{R}\in \left\{\frac{1}{n},\frac{2}{n},\cdots ,1\right\}\\ 0 & \hat{R}=0 \end{array}\right.\right\}\geq 1-\delta ,\\ \; & P\left\{R\leq \left\{\begin{array}{cc} F^{-1}\left(1-\delta ;n\hat{R}+1,n-n\hat{R}\right) & \hat{R}\in \left\{0,\frac{1}{n},\cdots ,\frac{n-1}{n}\right\}\\ 1 & \hat{R}=1 \end{array}\right.\right\}\geq 1-\delta . \end{array}$$

**Theorem** **A3** ([35]). *If $n\in N^{*}$, the following bounds hold*

$$\begin{array}{c} \sqrt{2\pi n}n^{n}e^{-n}\leq n!\leq \sqrt{2\pi n}n^{n}e^{-n}e^{\frac{1}{12n}}. \end{array}$$

**Theorem** **A4** ([6]). *If $n\in N^{*}$, p∈[0,1], q∈{0,1n,2n,⋯,1}, and $\hat{R}<p$, the following bound holds*

$$\begin{array}{c} F\left(p;nq,n-nq+1\right)\leq e^{-n\mathrm{kl}(q||p)} \end{array}$$

*where*

Fp;nq,n−nq+1=∑i=0nqnipi(1−p)n−i,

*is the beta cumulative density function, and*

$$\begin{array}{c} \mathrm{kl}\left(q||p\right)=q\ln\left(\frac{q}{p}\right)+\left(1-q\right)\ln\left(\frac{1-q}{1-p}\right) \end{array}$$

*is the Kullback–Leibler divergence.*

## Appendix B. Proofs

Proofs of our results are included in this section.

**Proof.** **(Lemma 2)** Please note that under the assumptions of the lemma, $\forall r_{1},\cdots ,r_{m}\in \left[0,1\right]$, and $\forall i\in I\backslash i^{*}$ and $\forall r_{k}^{\prime },r_{k}^{\prime \prime }\in \left[0,1\right]$ such that $r_{k}^{\prime }<r_{k}^{\prime \prime }$

$$\begin{array}{c} \sqrt{\frac{\log\left(\frac{2\sum_{j\in I\backslash k}e^{-\gamma \max[\theta ,r_{j}]}+e^{-\gamma \max[\theta ,r_{k}^{\prime }]}}{\delta e^{-\gamma \max[\theta ,r_{i}]}}\right)}{2n}}-\sqrt{\frac{\log\left(\frac{2\sum_{j\in I\backslash k}e^{-\gamma \max[\theta ,r_{j}]}+e^{-\gamma \max[\theta ,r_{k}^{\prime \prime }]}}{\delta e^{-\gamma \max[\theta ,r_{i}]}}\right)}{2n}}\geq 0, \end{array}$$

then the statement of the lemma is proved. □

**Proof.** **(Lemma 3)** Please note that under the assumptions of the lemma

$$\begin{array}{c} 0<\min\left[1,{\hat{R}}_{i}+\sqrt{\frac{\log\left(\frac{2\sum_{j\in I}e^{-\gamma \max[\theta ,r_{j}]}}{\delta e^{-\gamma \max[\theta ,r_{i^{*}}]}}\right)}{2n}}\right]\leq 1. \end{array}$$

Note also that if

$$\begin{array}{cc} \; & \gamma \leq 2\sqrt{n}\\ \; & p_{i^{*}}=\frac{e^{-\gamma r_{i^{*}}}}{\sum_{j\in I}e^{-\gamma r_{j}}} \end{array}$$

then

$$\begin{array}{c} \frac{\partial \sqrt{\frac{\log\left(\frac{2\sum_{j\in I}e^{-\gamma r_{j}}}{\delta e^{-\gamma r_{i^{*}}}}\right)}{2n}}}{\partial r_{i^{*}}}=\frac{\gamma p_{i^{*}}\left(1-p_{i^{*}}\right)}{4np_{i^{*}}\sqrt{\frac{\ln\left(\frac{2}{\delta p_{i^{*}}}\right)}{2n}}}<\frac{\left(1-p_{i^{*}}\right)}{2\sqrt{\frac{\ln\left(\frac{2}{\delta p_{i^{*}}}\right)}{2}}}<1, \end{array}$$

then the statement of the lemma is proved. □

**Proof.** **(Theorem 4)** Let us define

$$\begin{array}{c} \vartheta =\min\left[1,{\hat{R}}_{i^{*}}+\sqrt{\frac{\log\left(\frac{2m}{\delta }\right)}{2n}}\right]. \end{array}$$

Let us suppose that

$$\begin{array}{c} r_{i}\leq \vartheta ,\;\forall i\in I\backslash i^{*}. \end{array}$$

If we set

$$\begin{array}{c} r_{i^{*}}^{*}=\vartheta , \end{array}$$

we have, thanks to the hypothesis of the theorem that

$$\begin{array}{c} \frac{e^{-\gamma \max[\theta ,r_{i}]}}{\sum_{j\in I}e^{-\gamma \max[\theta ,r_{j}]}}=\frac{1}{m},\;\forall i\in I, \end{array}$$

then $r_{i^{*}}^{*}$ is a fixed point and since it is unique by Lemma 3 we have that $r_{i^{*}}^{*}\leq \vartheta$. Let us suppose now that

$$\begin{array}{c} r_{i}\leq \vartheta ,\;\forall i\in I\backslash \left\{i^{*},k\right\},\;r_{k}>\vartheta ,\;k\in I\backslash i^{*}. \end{array}$$

Thanks to the hypothesis of the theorem, we can state that

$$\begin{array}{c} \frac{e^{-\gamma \max[\theta ,r_{i^{*}}]}}{\sum_{j\in I\backslash \{i^{*},k\}}e^{-\gamma \max[\theta ,r_{j}]}+e^{-\gamma \max[\theta ,r_{i^{*}}]}+e^{-\gamma \max[\theta ,r_{k}]}}>\frac{e^{-\gamma \max[\theta ,r_{i^{*}}]}}{\sum_{j\in I\backslash i^{*}}e^{-\gamma \theta }+e^{-\gamma \max[\theta ,r_{i^{*}}]}}, \end{array}$$

and, consequently, we can also state that

$$\begin{array}{cc} r_{i^{*}}\; & =\min\left[1,{\hat{R}}_{i^{*}}+\sqrt{\frac{\log\left(\frac{2\sum_{j\in I\backslash \{i^{*},k\}}e^{-\gamma \max[\theta ,r_{j}]}+e^{-\gamma \max[\theta ,r_{i^{*}}]}+e^{-\gamma \max[\theta ,r_{k}]}}{\delta e^{-\gamma \max[\theta ,r_{i^{*}}]}}\right)}{2n}}\right]\\ \; & \leq \min\left[1,{\hat{R}}_{i^{*}}+\sqrt{\frac{\log\left(\frac{2\sum_{j\in I\backslash i^{*}}e^{-\gamma \theta }+e^{-\gamma \max[\theta ,r_{i^{*}}]}}{\delta e^{-\gamma \max[\theta ,r_{i^{*}}]}}\right)}{2n}}\right]. \end{array}$$

Consequently, by exploiting the same reasoning exploited before, $r_{i^{*}}^{*}\leq \vartheta$. By induction, the statement of the theorem is proved. □

**Proof.** **(Lemma 4)** Please note that

$$\begin{array}{c} \min\left[R_{1},\cdots ,R_{m}\right]\geq \max\left[0,\min\left[{\hat{R}}_{1},\cdots ,{\hat{R}}_{m}\right]-\sqrt{\frac{\log\left(\frac{2m}{\delta }\right)}{2n}}\right]. \end{array}$$

Then we can state that

$$\begin{array}{c} {\hat{R}}_{i^{*}}\leq \min\left[R_{1},\cdots ,R_{m}\right]+\sqrt{\frac{\log\left(\frac{2m}{\delta }\right)}{2n}}, \end{array}$$

and consequently, the statement of the theorem is proved. □

**Proof.** **(Theorem 5)** Let us define

$$\begin{array}{c} \vartheta =U\left({\hat{R}}_{i},\frac{\delta }{2m}\right). \end{array}$$

Let us suppose that

$$\begin{array}{c} r_{j}\leq \vartheta ,\;\forall j\in I\backslash i. \end{array}$$

If we set

$$\begin{array}{c} r_{i}^{*}=\vartheta , \end{array}$$

we have, thanks to the hypothesis of the theorem that

$$\begin{array}{c} f_{1}\left(r_{1},\cdots ,r_{i-1},r_{i}^{*},r_{i+1},\cdots ,r_{m}\right)=\cdots =f_{m}\left(r_{1},\cdots ,r_{i-1},r_{i}^{*},r_{i+1},\cdots ,r_{m}\right)=\frac{1}{m}, \end{array}$$

then $r_{i}^{*}$ is a fixed point and since it is unique by Lemma 7 we have that $r_{i}^{*}\leq \vartheta$. Let us suppose now that

$$\begin{array}{c} r_{j}\leq \vartheta ,\;\forall j\in I\backslash \left\{i,k\right\},\;r_{k}>\vartheta ,\;k\in I\backslash i. \end{array}$$

Thanks to the hypothesis of the theorem, we can state that

$$\begin{array}{c} f_{i}\left(\vartheta ,\cdots ,\vartheta ,r_{i},\vartheta ,\cdots ,\vartheta ,r_{k},\vartheta ,\cdots ,\vartheta \right)>f_{i}\left(\vartheta ,\cdots ,\vartheta ,r_{i},\vartheta ,\cdots ,\vartheta \right), \end{array}$$

and, consequently, we can also state that

$$\begin{array}{cc} r_{i} & =U\left({\hat{R}}_{i},\frac{\delta f_{i}\left(\vartheta ,\cdots ,\vartheta ,r_{i},\vartheta ,\cdots ,\vartheta ,r_{k},\vartheta ,\cdots ,\vartheta \right)}{2}\right)\\ \; & \leq U\left({\hat{R}}_{i},\frac{\delta f_{i}\left(\vartheta ,\cdots ,\vartheta ,r_{i},\vartheta ,\cdots ,\vartheta \right)}{2}\right). \end{array}$$

Consequently, by exploiting the same reasoning exploited before, $r_{i}^{*}\leq \vartheta$. By induction, the statement of the theorem is proved. □

**Proof.** **(Lemma 8)** Please note that

$$\begin{array}{c} \min\left[R_{1},\cdots ,R_{m}\right]\geq L\left(\min\left[{\hat{R}}_{1},\cdots ,{\hat{R}}_{m}\right],\frac{\delta }{2m}\right). \end{array}$$

Then we can state that

$$\begin{array}{c} {\hat{R}}_{i^{*}}\leq L^{-1}\left(\min\left[R_{1},\cdots ,R_{m}\right],\frac{\delta }{2m}\right), \end{array}$$

and consequently, the statement of the theorem is proved. □

**Proof.** **(Lemma 9)** To prove the theorem we first just have to note that for a fixed θ, the problem can be solved with Algorithm 1 and that its solution is $r_{i^{*}}^{*}\left(\theta \right)$ and $r_{j}\left(\theta \right)=L\left({\hat{R}}_{j},\frac{\delta }{2m}\right)$ with $j\in I\backslash i^{*}$. Then, searching for the largest fixed point, varying θ, such that

$$\begin{array}{c} \theta =U\left(L^{-1}\left(\min\left[r_{1}\left(\theta \right),\cdots ,r_{i^{*}-1}\left(\theta \right),r_{i^{*}}^{*}\left(\theta \right),r_{i^{*}+1}\left(\theta \right),\cdots ,r_{m}\left(\theta \right)\right],\frac{\delta }{2m}\right),\frac{\delta }{2m}\right) \end{array}$$

gives the proposed algorithm. □

**Proof.** **(Lemma 10)** For what concerns Corollary 2, we must prove that $\forall r_{1},\cdots ,r_{m}\in \left[0,1\right]$

$$\begin{array}{c} f_{i}\left(r_{1},\cdots ,r_{m}\right)\in \left(0,1\right)\;\forall i\in I,\;\sum_{i\in I}f_{i}\left(r_{1},\cdots ,r_{m}\right)=1. \end{array}$$

These two properties are trivially provable by definition. For what concerns Theorem 5, instead, we must prove that for θ∈[0,1], $\forall r_{1},\cdots ,r_{m}\in \left[0,1\right]$, and $\forall r_{j}^{\prime },r_{j}^{\prime \prime }\in \left[0,\theta \right]$

$$\begin{array}{c} f_{j}\left(r_{1},\cdots ,r_{j-1},r_{j}^{\prime },r_{j+1},\cdots ,r_{m}\right)-f_{j}\left(r_{1},\cdots ,r_{j-1},r_{j}^{\prime \prime },r_{j+1},\cdots ,r_{m}\right)=0, \end{array}$$

with j∈I. Moreover, we must prove that $\forall r_{1},\cdots ,r_{m}\in \left[0,\theta \right]$ and ∀j∈I

$$\begin{array}{c} f_{j}\left(r_{1},\cdots ,r_{m}\right)=\frac{1}{m}. \end{array}$$

These properties are trivially provable by definition. Then we must prove that $\forall r_{1},\cdots ,r_{m}\in \left[0,1\right]$, ∀j∈I\i, $\forall r_{j}^{\prime },r_{j}^{\prime \prime }\in \left(\theta ,1\right]$ such that $r_{j}^{\prime }<r_{j}^{\prime \prime }$

$$\begin{array}{c} f_{i}\left(r_{1},\cdots ,r_{j-1},r_{j}^{\prime },r_{j+1},\cdots ,r_{m}\right)-f_{i}\left(r_{1},\cdots ,r_{j-1},r_{j}^{\prime \prime },r_{j+1},\cdots ,r_{m}\right)<0. \end{array}$$

Then, let us note that

$$\begin{array}{cc} \; & \frac{e^{-\gamma \max[\theta ,r_{i}]}}{e^{-\gamma \max[\theta ,r_{j}^{\prime \prime }]}+\sum_{k\in I\backslash j}e^{-\gamma \max[\theta ,r_{k}]}}-\frac{e^{-\gamma \max[\theta ,r_{i}]}}{e^{-\gamma \max[\theta ,r_{j}^{\prime }]}+\sum_{k\in I\backslash j}e^{-\gamma \max[\theta ,r_{k}]}}\\ \; & >\frac{e^{-\gamma \max[\theta ,r_{i}]}}{e^{-\gamma \max[\theta ,r_{j}^{\prime }]}+\sum_{k\in I\backslash j}e^{-\gamma \max[\theta ,r_{k}]}}-\frac{e^{-\gamma \max[\theta ,r_{i}]}}{e^{-\gamma \max[\theta ,r_{j}^{\prime }]}+\sum_{k\in I\backslash j}e^{-\gamma \max[\theta ,r_{k}]}}=0, \end{array}$$

consequently, the property is proven. □

**Proof.** **(Lemma 11)** For what concerns Corollary 2 and Theorem 5, the proof is a trivial consequence of Lemma 10. For what concerns Lemma 6, we have to prove that ∀r^i∈0,1m,⋯,1, $\forall r_{1},\cdots ,r_{m}\in \left[0,1\right]$, and ∀j∈I\i and $\forall r_{j}^{\prime },r_{j}^{\prime \prime }\in \left[0,1\right]$ such that $r_{j}^{\prime }<r_{j}^{\prime \prime }$

$$\begin{array}{c} U\left({\hat{r}}_{i},\frac{\delta f_{i}\left(r_{1},\cdots ,r_{j-1},r_{j}^{\prime },r_{j+1},\cdots ,r_{m}\right)}{2}\right)-U\left({\hat{r}}_{i},\frac{\delta f_{i}\left(r_{1},\cdots ,r_{j-1},r_{j}^{\prime \prime },r_{j+1},\cdots ,r_{m}\right)}{2}\right)\geq 0, \end{array}$$

where i∈I. Let us define

$$\begin{array}{c} g_{i}\left(r_{1},\cdots ,r_{m}\right)=\frac{e^{-\gamma r_{i}}}{\sum_{j\in I}e^{-\gamma r_{j}}},\;i\in I, \end{array}$$

and note that

$$\begin{array}{c} \frac{\partial g_{i}\left(r_{1},\cdots ,r_{m}\right)}{\partial r_{j}}=\gamma g_{i}\left(r_{1},\cdots ,r_{m}\right)g_{j}\left(r_{1},\cdots ,r_{m}\right),\;i\in I,\;j\in I\backslash i. \end{array}$$

Combining this property with Proposition A1 we obtain the result. For what concerns Lemma 7, we must prove that $\forall r_{i}^{\prime },r_{i}^{\prime \prime }\in \left[0,1\right]$ such that $r_{i}^{\prime }<r_{i}^{\prime \prime }$

$$\begin{array}{cc} \; & \frac{U\left({\hat{R}}_{i},\frac{\delta f_{i}\left(r_{1},\cdots ,r_{i}^{\prime \prime },\cdots ,r_{m}\right)}{2}\right)-U\left({\hat{R}}_{i},\frac{\delta f_{i}\left(r_{1},\cdots ,r_{i}^{\prime },\cdots ,r_{m}\right)}{2}\right)}{r_{i}^{\prime \prime }-r_{i}^{\prime }}<1,\\ \; & U\left({\hat{R}}_{i},\frac{\delta f_{i}\left(r_{1},\cdots ,r_{i-1},0,r_{i+1},\cdots ,r_{m}\right)}{2}\right)>0,\\ \; & U\left({\hat{R}}_{i},\frac{\delta f_{i}\left(r_{1},\cdots ,r_{i-1},1,r_{i+1},\cdots ,r_{m}\right)}{2}\right)<1. \end{array}$$

The second and the third properties are trivially true if ${\hat{R}}_{i}\neq 1$. For the first, instead, note that

$$\begin{array}{c} \frac{\partial g_{i}\left(r_{1},\cdots ,r_{m}\right)}{\partial r_{i}}=-\gamma g_{i}\left(r_{1},\cdots ,r_{m}\right)\left(1-g_{i}\left(r_{1},\cdots ,r_{m}\right)\right),\;i\in I. \end{array}$$

Then we can state, thanks also to Proposition A1 that

$$\begin{array}{cc} \; & \frac{U\left({\hat{R}}_{i},\frac{\delta f_{i}\left(r_{1},\cdots ,r_{i}^{\prime \prime },\cdots ,r_{m}\right)}{2}\right)-U\left({\hat{R}}_{i},\frac{\delta f_{i}\left(r_{1},\cdots ,r_{i}^{\prime },\cdots ,r_{m}\right)}{2}\right)}{r_{i}^{\prime \prime }-r_{i}^{\prime }}\\ \; & \leq \max_{\hat{r}\in \left\{0,\frac{1}{n},\cdots ,\frac{n-1}{n}\right\},\;p\in \left(0,1\right)}\frac{B(n\hat{r}+1,n-n\hat{r})}{{\left(U\left(\hat{r},\frac{\delta p}{2}\right)\right)}^{n\hat{r}}{\left(1-U\left(\hat{r},\frac{\delta p}{2}\right)\right)}^{n-n\hat{r}-1}}\frac{\delta }{2}\gamma p\left(1-p\right) \end{array}$$

Since this quantity must be strictly smaller than one we have that

$$\begin{array}{c} \gamma <\min_{\hat{r}\in \left\{0,\frac{1}{n},\cdots ,\frac{n-1}{n}\right\},\;p\in \left(0,1\right)}\frac{{\left(U\left(\hat{r},\frac{\delta p}{2}\right)\right)}^{n\hat{r}}{\left(1-U\left(\hat{r},\frac{\delta p}{2}\right)\right)}^{n-n\hat{r}-1}}{B\left(n\hat{r}+1,n-n\hat{r}\right)\frac{\delta }{2}p\left(1-p\right)}, \end{array}$$

Note also that by denoting

$$\begin{array}{c} \hat{s}=n\hat{r},\;U=U\left(\hat{r},\frac{\delta p}{2}\right), \end{array}$$

then, thanks to Theorems A3 and A4 we can state that

B(s^+1,n−s^)1Us^1−Un−s^−1p=s^!(n−s^−1)!n!1Us^1−Un−s^−1p≤2πs^s^s^e−s^e112s^2π(n−s^−1)(n−s^−1)n−s^−1e−(n−s^−1)e112(n−s^−1)2πnnne−n1Us^1−Un−s^−1p=2πe1+112s^+112(n−s^−1)s^s^s^(n−s^−1)(n−s^−1)n−s^−1nnn1Us^1−Un−s^−1p≤2πe76s^(n−s^)s^s^(n−s^)n−s^nnn1Us^1−Un−s^1−Un−s^p=2πe76s^(n−s^)s^s^(n−s^)n−s^nnn1Us^1−Un−s^1−Un1−s^np≤2πe76s^(n−s^)s^s^(n−s^)n−s^nnn1Us^1−Un−s^1np=2πe76s^(n−s^)n1e−ns^nlns^nU+n−s^nln1−s^n1−U1np≤2πe76s^(n−s^)n1∑i=0s^niUi(1−U)n−i1np=2πe76s^n1−s^nn2δ≤2πe761n1δ,

and, consequently

$$\begin{array}{c} \min_{\hat{r}\in \left\{0,\frac{1}{n},\cdots ,\frac{n-1}{n}\right\},\;p\in \left(0,1\right)}\frac{{\left(U\left(\hat{r},\frac{\delta p}{2}\right)\right)}^{n\hat{r}}{\left(1-U\left(\hat{r},\frac{\delta p}{2}\right)\right)}^{n-n\hat{r}-1}}{B\left(n\hat{r}+1,n-n\hat{r}\right)\frac{\delta }{2}p\left(1-p\right)}\geq \frac{2\sqrt{n}}{\sqrt{2\pi }e^{\frac{7}{6}}}. \end{array}$$

Finally, for what concerns Lemma 8, we must prove that ∀r^,r^′,r^″∈{0,1m,⋯,1} such that ${\hat{r}}^{\prime }<{\hat{r}}^{\prime \prime }$, we have that

$$\begin{array}{cc} \; & L\left({\hat{r}}^{\prime },\delta \right)-L\left({\hat{r}}^{\prime \prime },\delta \right)\leq 0,\\ \; & \exists L^{-1}:L^{-1}\left(L\left(\hat{r},n,\delta \right),\delta \right)\geq \hat{r}. \end{array}$$

Exploiting Proposition A1 the first property can be easily derived. The second property is true by definition of $L^{-1}\left(r,\delta \right)$. □

## Appendix C. Technicalities

Technicalities of the paper are included in this section.

**Proposition** **A1** (for Theorem A2). *Let us define*

$$\begin{array}{cc} \; & L\left(\hat{R},n,\delta \right)=\left\{\begin{array}{cc} F^{-1}\left(\delta ;n\hat{R},n-n\hat{R}+1\right) & \hat{R}\in \left\{\frac{1}{n},\frac{2}{n},\cdots ,1\right\}\\ 0 & \hat{R}=0 \end{array}\right.,\\ \; & U\left(\hat{R},n,\delta \right)=\left\{\begin{array}{cc} F^{-1}\left(1-\delta ;n\hat{R}+1,n-n\hat{R}\right) & \hat{R}\in \left\{0,\frac{1}{n},\cdots ,\frac{n-1}{n}\right\}\\ 1 & \hat{R}=1 \end{array}\right., \end{array}$$

*then*

$$\begin{array}{c} L\left(\hat{R},n,\delta \right)\in \left[0,1\right],\;U\left(\hat{R},n,\delta \right)\in \left[0,1\right], \end{array}$$

*and*

$$\begin{array}{cc} \; & \frac{\partial L(\hat{R},\delta )}{\partial \delta }=\left\{\begin{array}{cc} \frac{B(n\hat{R},n-n\hat{R}+1)}{{\left(L\left(\hat{R},\delta \right)\right)}^{n\hat{R}-1}{\left(1-L\left(\hat{R},\delta \right)\right)}^{n-n\hat{R}}}\; & \hat{R}\in \left\{\frac{1}{n},\frac{2}{n},\cdots ,1\right\}\\ 0 & \hat{R}=0 \end{array}\right.\in \left[0,\infty \right),\\ \; & \frac{\partial U(\hat{R},\delta )}{\partial \delta }=\left\{\begin{array}{cc} \frac{-B(n\hat{R}+1,n-n\hat{R})}{{\left(U\left(\hat{R},\delta \right)\right)}^{n\hat{R}}{\left(1-U\left(\hat{R},\delta \right)\right)}^{n-n\hat{R}-1}}\; & \hat{R}\in \left\{0,\frac{1}{n},\cdots ,\frac{n-1}{n}\right\}\\ 0 & \hat{R}=1 \end{array}\right.\in \left(-\infty ,0\right]. \end{array}$$

The derivation of Proposition A1 is trivial.

## Appendix D. Code

All Matlab codes used in this paper are available by request to the corresponding author.
